# Targeting Aromatase and HDACs in Hormone Receptor-Positive Breast Cancer: Molecular Interplay, Endocrine Resistance and Therapeutic Opportunities

**DOI:** 10.3390/ijms27198619

**Published:** 2026-09-26

**Authors:** Roxana Liana Lucaciu, Adriana Corina Hangan, Luminița Simona Oprean, Lucia Maria Procopciuc

**Affiliations:** 1 Department of Pharmaceutical Biochemistry and Clinical Laboratory, Faculty of Pharmacy, “Iuliu-Hațieganu” University of Medicine and Pharmacy, 400349 Cluj-Napoca, Romania; liana.lucaciu@umfcluj.ro; 2 Department of Inorganic Chemistry, Faculty of Pharmacy, “Iuliu-Hațieganu” University of Medicine and Pharmacy, 400012 Cluj-Napoca, Romania; adriana.hangan@umfcluj.ro; 3 Department of Medical Biochemistry, Faculty of Medicine, “Iuliu Hațieganu” University of Medicine and Pharmacy, 400349 Cluj-Napoca, Romania; lprocopciuc@umfcluj.ro

**Keywords:** breast cancer, estrogen receptor-positive breast cancer, aromatase inhibitors, HDAC inhibitors

## Abstract

Breast cancer is the most frequently diagnosed malignancy in women, and hormone receptor-positive disease represents its predominant subtype. Aromatase inhibitors (AIs) are a cornerstone of endocrine therapy, particularly in postmenopausal patients, but their long-term efficacy is frequently limited by intrinsic or acquired resistance. Increasing evidence indicates that epigenetic dysregulation, especially aberrant histone deacetylase (HDAC) activity, contributes to altered estrogen receptor signaling, transcriptional plasticity, activation of compensatory survival pathways, and endocrine treatment failure. This narrative review examines the biological and therapeutic interplay between aromatase and HDACs in breast cancer, integrating evidence from mechanistic studies, in vitro models, preclinical experiments, and clinical trials. HDAC inhibition may restore endocrine sensitivity by modifying chromatin accessibility, reactivating estrogen-responsive and tumor-suppressor genes, suppressing adaptive pathways such as HER2, NF-κB, and PI3K/AKT/mTOR signaling, and promoting cell-cycle arrest and apoptosis. Preclinical studies support synergistic or sensitizing effects of combined AI–HDAC inhibition, while clinical trials with entinostat, tucidinostat, panobinostat, and exemestane- or letrozole-based regimens have produced heterogeneous results. These findings suggest that endocrine–epigenetic combinations may benefit selected patients rather than represent an universally effective strategy. Future clinical translation will require improved HDAC selectivity, optimized dosing, toxicity management, and validation of predictive biomarkers enabling biomarker-guided patient selection.

## 1. Introduction

Human carcinogenesis is a multistage, multifactorial process, occurring at both phenotypic and genetic levels, reflecting the genetic alterations that lead to the progressive transformation of a normal cell into a malignant one. Phenotypic changes are progressive, continuously multiplying and occurring in an uncontrolled manner. The development of the malignant phenotype results from multiple interactions between various exogenous and endogenous factors (genetic, hormonal, immunological, and metabolic). The carcinogenic process is long-term and occurs through multiple stages, including tumor initiation, tumor promotion, malignant transformation, and tumor progression. The latter results from the accumulation of genetic and epigenetic alterations [1,2]. Genomic instability predisposes to malignant dissemination and may occur at both macro- and microsomal levels (replication fidelity, repair defects, deletions, insertions, amplifications, etc.). Mutations in genes such as p53, Rb, or DNA repair genes can increase the mutation rate of other genes, resulting in a mutator phenotype [3]. Epigenetic changes, such as alterations in DNA methylation or acetylation status, lead to the suppression or activation of genes in specific regions [4,5].

From a global health perspective, breast cancer represents the most commonly diagnosed malignancy among women worldwide and remains a major cause of cancer-related mortality. According to the International Agency for Research on Cancer (IARC), the cancer agency of the World Health Organization (WHO), approximately 2.3 million new cases of female breast cancer and about 670,000 related deaths occurred globally in 2022. If current incidence and mortality rates persist, the annual burden is projected to reach approximately 3.2 million new cases and 1.1 million breast cancer-related deaths by 2050, corresponding to increases of approximately 38% and 68%, respectively. These projections emphasize the growing global impact of breast cancer and the continuing need for improved prevention, early detection, and therapeutic strategies [6,7].

Breast cancer development is influenced by both genetic and hormonal factors. Pathogenic alterations in high-penetrance susceptibility genes, particularly breast cancer gene 1 (BRCA1), BRCA2, and tumor protein p53 (TP53), are associated with an increased risk of breast cancer, while prolonged estrogen exposure also contributes to breast carcinogenesis. Aromatase, which catalyzes the final step in estrogen biosynthesis, is therefore an important determinant of estrogen availability [8,9].

Beyond genetic alterations, carcinogenesis also involves epigenetic mechanisms, including DNA methylation and histone acetylation/deacetylation. Among the epigenetic alterations associated with cancer, aberrant deacetylation of histones H3 and H4 promotes chromatin condensation and transcriptional repression. These changes alter gene expression by silencing tumor suppressor genes and genes involved in DNA repair, apoptosis, and cell-cycle regulation. Consequently, enzymes involved in these epigenetic processes have emerged as important therapeutic targets in cancer. Histone acetylation, one of the best-characterized post-translational histone modifications, is dynamically regulated by the opposing activities of histone acetyltransferases (HATs) and histone deacetylases (HDACs). Dysregulation of this balance contributes to aberrant gene expression and tumor development [10].

HDACs regulate chromatin accessibility and interact with several nuclear receptors, including estrogen receptors (ERs), thereby modulating estrogen-responsive gene transcription, cellular proliferation, differentiation, and survival. ERα, the predominant estrogen receptor isoform expressed in breast cancer, is a member of the nuclear receptor superfamily and regulates the expression of genes essential for mammary gland development and function. Among HDAC family members, HDAC1 has been shown to interact with ERα, suppressing its transcriptional activity and contributing to the epigenetic regulation of estrogen-responsive gene expression [11,12].

Among the different molecular subtypes, hormone receptor-positive (HR+) breast cancer, defined by the expression of ER and/or progesterone receptor (PR) in ≥1% of tumor cells, represents the most prevalent subtype [13]. The management of breast cancer requires an individualized and balanced therapeutic approach, integrating multiple treatment modalities according to tumor characteristics and patient risk. The current therapeutic armamentarium includes surgery, neoadjuvant and adjuvant chemotherapy, endocrine therapy, targeted therapies, and other systemic treatment strategies. In particular, endocrine therapy with selective estrogen receptor modulators (SERMs), such as tamoxifen, and aromatase inhibitors (AIs) represents a cornerstone of treatment for patients with HR+ breast cancer [14]. Endocrine therapy is generally administered for a minimum of five years, although treatment duration may be extended in selected patients according to their risk of recurrence and tolerability [15]. Increasing evidence supports extending endocrine therapy beyond five years, particularly among patients with a higher baseline risk of late disease recurrence [16].

Although aromatase and histone deacetylases belong to distinct biological pathways, increasing evidence indicates a significant functional interplay between estrogen biosynthesis and epigenetic regulation. HDACs modulate estrogen receptor signaling, influence the expression of genes involved in estrogen-dependent proliferation, and contribute to the development of endocrine resistance. Conversely, estrogen signaling may regulate the expression and activity of several epigenetic modifiers. Therefore, simultaneously targeting aromatase and HDACs has emerged as a promising therapeutic strategy capable of enhancing endocrine responsiveness and overcoming resistance in hormone receptor-positive breast cancer [17,18].

Unlike previous reviews that have largely addressed aromatase inhibition, endocrine resistance, or HDAC-targeted therapy as separate topics, the present review integrates these areas within a unified mechanistic and translational framework.

Particular emphasis is placed on the functional interplay between estrogen biosynthesis, ER-dependent transcription, and HDAC-mediated epigenetic regulation, and on how these interconnected processes contribute to acquired resistance to AIs. Furthermore, the review critically links mechanistic findings with in vitro, preclinical in vivo, and clinical evidence for the combined targeting of aromatase-dependent estrogen signaling and HDAC-mediated epigenetic regulation, highlighting both the biological rationale for this therapeutic strategy and the heterogeneity of clinical responses. This integrated perspective may help identify molecular determinants of therapeutic sensitivity and support the development of biomarker-guided endocrine–epigenetic combinations in HR+ breast cancer.

The aim of this narrative review is to provide an integrated overview of the functional interplay between aromatase-dependent estrogen signaling and HDAC-mediated epigenetic regulation in breast cancer, with particular emphasis on their convergence in mechanisms of endocrine resistance and on the therapeutic rationale for combining aromatase inhibitors with HDACi. In addition, the review critically summarizes the in vitro, preclinical, and clinical evidence supporting this endocrine–epigenetic therapeutic strategy in HR+ breast cancer. This narrative review was based on a structured literature search performed in PubMed/MEDLINE, Scopus, and Web of Science for articles published up to June 2026. The search strategy included combinations of the following terms: “breast cancer”, “estrogen receptor-positive breast cancer”, “HER2-negative breast cancer”, “aromatase”, “aromatase inhibitors”, “histone deacetylases”, “HDAC inhibitors”, “endocrine resistance”, “epigenetic regulation”, and “molecular mechanisms”. Original experimental studies, in vitro and in vivo studies, clinical trials, observational studies, systematic reviews, meta-analyses, and relevant narrative reviews were considered. Particular attention was given to studies investigating the interaction between AI and HDAC inhibition in estrogen receptor-positive (ER+)/human epidermal growth factor receptor 2 (HER2)-negative breast cancer and in models of acquired resistance to AIs. Studies unrelated to breast cancer or without relevance to aromatase, HDAC biology, endocrine resistance, or combined AI–HDAC inhibition were excluded. Reference lists of selected publications were also screened manually to identify additional relevant studies. The evidence was synthesized narratively and organized into mechanistic, in vitro, preclinical in vivo, and clinical findings.

Although AIs remain the cornerstone of endocrine therapy for HR+ breast cancer, acquired resistance eventually develops in a substantial proportion of patients. Increasing evidence suggests that epigenetic alterations mediated by HDACs contribute to endocrine resistance and disease progression. Consequently, combined targeting of aromatase and HDACs has attracted considerable attention as a novel therapeutic strategy [17,18].

## 2. Estrogens and Aromatase as Therapeutic Targets in Breast Cancer

ER+ breast cancer represents the most common molecular subtype of breast cancer, accounting for approximately 70–80% of all cases [19]. It is characterized by the expression of ERs on tumor cells, which, upon activation by estrogens, promote cellular proliferation and tumor growth [20]. The pathophysiology of ER+ breast cancer is multifactorial and involves complex interactions between hormonal signaling, the tumor microenvironment, and genetic alterations. Estrogen-mediated activation of ER signaling can stimulate aberrant cell proliferation, while interactions between tumor cells and the surrounding microenvironment may further enhance hormone-dependent signaling. In addition, acquired mutations in the estrogen receptor 1 gene (ESR1) can lead to constitutive or ligand-independent ER activation and are an important mechanism underlying resistance to endocrine therapy [21,22].

The main estrogens are estradiol, estrone, and 16-hydroxyestradiol (estriol), with estradiol being the most biologically active. Estrone, in turn, can be reversibly converted into estradiol under the action of a specific 17β-hydroxysteroid dehydrogenase. During the premenopausal period, the predominant estrogen is estradiol (synthesized in the ovaries), whereas in postmenopause, estrone (synthesized in peripheral tissues) becomes predominant [23].

### 2.1. Estrogens Metabolism and Carcinogenesis

Starting from estrogens, the mechanism of carcinogenesis essentially involves their metabolism, which unfortunately leads to genotoxic and mutagenic metabolites that stimulate tissue growth [24].

Both estradiol and estrone are metabolized through three competing metabolic pathways involving irreversible hydroxylation reactions catalyzed by enzymes belonging to the NADPH-dependent cytochrome P450 superfamily. The main isoforms involved are CYP1A1, CYP1B1, and CYP1A2. Hydroxylation occurs at positions C2, C4, and C16, respectively, leading to the conversion of estrogens into catechol estrogens: 2-hydroxyestrone, 4-hydroxyestrone, 2-hydroxyestradiol, 4-hydroxyestradiol, and 16-hydroxyestrone. Catechols are further metabolized through methylation at the same positions, generating the corresponding methoxyestrogens (e.g., 2-methoxyestrone) [25]. The enzyme involved in this process is catechol-O-methyltransferase (COMT). Under the action of peroxidases, semiquinones and quinones may be formed, which are reactive metabolic intermediates of redox cycling reactions and can cause DNA damage. These various metabolites can subsequently be conjugated with glucuronic acid or sulfate to form more polar, water-soluble compounds that are more easily excreted via the kidneys. The enzymes involved in these processes are UDP-glucuronosyltransferase and sulfotransferase, respectively [23] (Figure 1).

It is important to note that several in vitro studies have demonstrated that the 2-hydroxylated metabolites of estrone and estradiol inhibit the growth and proliferation of malignant cells. The lack of tumorigenic activity of 2-hydroxyl metabolites has been attributed, on one hand, to mechanisms involving a high clearance rate, and on the other hand, to a faster rate of *O*-methylation, leading to methoxy-type metabolites that suppress tumor cell proliferation and angiogenesis, particularly due to their effects on microtubule stabilization [26].

In contrast, 4-hydroxylated catechol estrogens have been shown to possess carcinogenic potential due to their ability to form depurinating adducts; the resulting apurinic sites generate mutations that may initiate carcinogenesis [27].

Finally, 16-hydroxyestrone has also been shown to be a potential tumor initiator, and increased serum levels have been associated with enhanced proliferation of malignant breast cells; thus, it may be considered a risk factor in breast cancer [28].

As mentioned above, the enzyme responsible for the key step in estrogen biosynthesis is aromatase.

Aromatase is a microsomal enzyme responsible for converting testosterone into estradiol and androstenedione into estrone. It corresponds to the CYP19A1 isoform, part of the cytochrome P450 superfamily of monooxygenases that catalyze key reactions in steroidogenesis. In humans, the enzyme is encoded by the *CYP19* gene, located on chromosome 15q21.1. It is found in tissues where estrogen is produced, including the adrenal glands, ovaries, breast, placenta, testes, adipose tissue (especially in postmenopausal women), brain, skin, and blood vessels. At the intracellular level, it is localized in the endoplasmic reticulum, where specific promoters regulating enzyme activity are also present; these are themselves controlled by hormones, cytokines, and other factors [29].

Aromatase acts in the final steps of conversion, involving three successive hydroxylations at the 19-methyl group of androgens, followed by its simultaneous removal as formyl, and the aromatization of the A ring (Figure 2) [23].

The catalytic mechanism of the aromatization reaction is a complex mechanism of sequential oxidation at the level of the methyl group attached to C19, requiring the simultaneous presence of molecular oxygen and a reduced cofactor (NADPH) [23].

An imbalance between estrogen production and metabolism—particularly the generation of quinones is considered a major factor in both the etiology and prevention of breast cancer. Studies have shown that women diagnosed with breast cancer, exhibit significantly higher levels of estrogen–DNA adducts compared to control groups. The same studies have revealed that specific antioxidants, such as N-acetylcysteine and resveratrol, can inhibit the oxidation of catechol estrogens into quinones and, consequently, their interaction with DNA, thereby acting as preventive factors in the initiation of carcinogenesis [30].

Aromatase plays a central role in the development and progression of estrogen-dependent breast cancer by catalyzing the local biosynthesis of estrogens in peripheral tissues, including breast tissue. In postmenopausal women, ovarian estrogen production declines substantially, making peripheral aromatization an important source of circulating and tissue estrogen. Accordingly, AIs are widely used to suppress estrogen biosynthesis and reduce estrogen receptor-mediated tumor growth in postmenopausal patients with HR+ breast cancer [31]. By selectively inhibiting the aromatase enzyme, AIs effectively decrease estrogen synthesis in peripheral tissues while avoiding direct suppression of ovarian function, which is particularly relevant in postmenopausal women [32].

The physiological contribution of aromatase to estrogen biosynthesis varies according to tissue and menopausal status. The major sites of aromatase expression, their contribution to estrogen production, principal estrogen-responsive tissues, and the differences between premenopausal and postmenopausal women are summarized in Table 1 [23,29,31,32].

Aromatase activity and estrogen availability can be influenced by several hormonal, metabolic, and tissue-specific factors, some of which may contribute to breast cancer risk, particularly in postmenopausal women [29,33].

Various factors, such as diet, can affect the activity of CYP1A1 and CYP1B1 enzymes and may indirectly lead to increased circulating estrogen levels. Recent literature places growing emphasis on nutritional factors, as many studies have demonstrated that they may have either negative or positive effects in the prevention or palliative treatment of various cancers [34]. In the case of breast cancer, studies suggest that low-fat and high-fiber diets may have a modest protective role; consumption of fruits and vegetables is not clearly associated with cancer risk, whereas alcohol consumption is positively associated with increased risk [35,36]. Other factors include: age—with risk increasing over time, doubling every 10 years until menopause; age at menarche; age at first pregnancy; age at menopause; family history; presence of previous benign tumors; geographical differences, highlighting the importance of environmental factors [37]. Another important factor is obesity, due to increased estrogen production via aromatase activation in adipose tissue at the level of the mammary gland [38].

Increased attention has also been given to genetic polymorphisms affecting estrogen metabolism (especially catechol estrogens) and to the relationship between their formation, their quinone metabolites, and breast cancer risk. The genotoxic potential of estrogens (through the metabolites and mechanisms described above) has been shown to vary between individuals and to be influenced by genetic variations caused by point mutations (resulting in single nucleotide polymorphisms). Studies have shown that certain allelic variants of *CYP1B1*, particularly *CYP1B1*2 and *CYP1B1*3, may be directly associated with cancer risk (although not in all cases), while the Met158 allele of COMT produces an enzyme with reduced functionality, thereby increasing the risk of quinone formation. The role of polymorphic forms of GST (glutathione S-transferase) is less clear, although a GST Val105 allele has been identified that produces an isoform with altered catalytic activity [39].

Another relatively recent aspect in identifying risk factors in breast carcinogenesis concerns a significant portion of the intestinal microbial flora, suggestively termed the estrobolome, and its function. The estrobolome is a subset of the microbiome essential for estrogen metabolism and regulation of the body’s estrogen load. Studies have shown that any imbalance and consequent dysfunction of this system may increase the risk of breast cancer recurrence in postmenopausal women with ER+ breast cancer. Its bacterial composition, which influences its function, is itself affected by factors such as age, ethnicity, diet, lifestyle, alcohol consumption, and antibiotic use. An enzyme has also been identified—β-glucuronidase (produced by pathogenic intestinal bacteria, particularly *Clostridium perfringens*)—which allows estrogens to re-enter the bloodstream and be recycled in the liver (enterohepatic recycling), thereby increasing estrogen levels [40].

In addition to endogenous estrogen levels, elevated serum levels of androstenediol and testosterone also represent risk factors, as these androgens can be converted into estrogens under the action of aromatase. Regarding the molecular mechanisms involved in breast carcinogenesis, it is important to highlight the existence of two distinct but complementary pathogenic pathways: the estrogen metabolite pathway (discussed above) and estrogen ER-mediated signaling [41]. Studies have shown that women who develop ER+ breast cancer are exposed to multiple risk factors, including age, duration of exposure to estrogen and progesterone (time between menarche and menopause), hormone therapy, obesity, and alcohol consumption. These two pathways may intersect, as catechol estrogen metabolites can be involved in controlling gene expression and/or signaling pathways mediated by ERs. The mechanisms by which estrogens contribute to each stage of carcinogenesis are complex. Studies suggest the involvement of genotoxic estrogen metabolites as well as genomic and non-genomic estrogen receptor-mediated signaling that affects cell proliferation and apoptosis in breast tissue. The way in which these two pathways contribute to estrogen-mediated carcinogenesis, as well as how genetic polymorphisms and environmental factors influence these processes, requires further investigation [42].

### 2.2. Obesity, Adipose Tissue Aromatase, and Estrogen Availability in Breast Cancer

Obesity, particularly increased adiposity after menopause, represents an important modifier of estrogen availability and breast cancer risk. Adipose tissue is not merely an energy-storage compartment but an active endocrine organ capable of producing estrogens through aromatase-dependent conversion of androgen precursors. Following menopause, when ovarian estrogen production markedly declines, peripheral adipose tissue becomes one of the major sites of estrogen biosynthesis. Consequently, women with increased body mass index (BMI) and greater adipose tissue mass may exhibit higher circulating and locally produced estrogen levels than lean postmenopausal women, providing a biologically plausible link between obesity and the development and progression of HR+ breast cancer [38,43,44].

The relationship between obesity and estrogen production is mediated not only by the greater amount of adipose tissue but also by obesity-associated changes within the adipose microenvironment. In obesity, adipocyte hypertrophy and adipose tissue dysfunction promote macrophage infiltration and chronic low-grade inflammation. These inflammatory changes are associated with increased production of cytokines and other mediators that can enhance CYP19A1 expression and aromatase activity in adipose stromal cells, thereby favoring the local conversion of androgen precursors to estrogens and increasing estrogen bioavailability within breast tissue [44,45].

Obesity may further enhance estrogen-dependent tumorigenic signaling through metabolic and endocrine alterations, including hyperinsulinemia, insulin resistance, and dysregulated adipokine signaling. These pathways can interact with ER and growth-factor signaling and contribute to a proliferative and inflammatory tumor microenvironment. Such mechanisms are particularly relevant in postmenopausal ER+ breast cancer, in which peripheral estrogen synthesis and obesity-associated metabolic dysfunction may cooperate in supporting tumor growth and progression [43,45].

BMI may also influence the therapeutic context of AI. Obesity and insulin resistance have been associated with poorer outcomes in ER+ breast cancer and may contribute to reduced endocrine responsiveness through several mechanisms, including persistent estrogenic stimulation and activation of compensatory growth and survival pathways. However, the extent to which increased BMI directly reduces the pharmacological efficacy of AIs remains incompletely defined and may vary according to clinical setting and treatment regimen [43].

Taken together, these findings indicate that obesity should be considered not only a conventional breast cancer risk factor but also an endocrine and metabolic modifier capable of influencing estrogen availability, tumor biology, and potentially the response to endocrine therapy [38,43,44].

### 2.3. Sex Hormone-Binding Globulin and Estrogen Bioavailability in Breast Cancer

Sex hormone-binding globulin (SHBG) is a circulating glycoprotein synthesized predominantly by hepatocytes and represents an important regulator of sex steroid transport and bioavailability. In the circulation, estrogens and androgens are largely protein-bound, principally to SHBG and albumin, whereas only a small unbound fraction is considered freely available for diffusion into target tissues. Consequently, circulating SHBG concentrations influence the proportion of biologically available estrogens and therefore may modulate estrogen-dependent signaling in hormone-responsive tissues [46].

The relationship between SHBG and breast cancer is particularly relevant in postmenopausal women, in whom ovarian estrogen production declines and peripheral estrogen synthesis becomes increasingly important. Lower circulating SHBG concentrations are associated with higher levels of free or bioavailable estrogens, potentially increasing estrogen receptor activation in breast tissue. Conversely, higher SHBG concentrations may reduce the biologically available fraction of estradiol and have been associated with a lower risk of postmenopausal ER+ breast cancer [46,47]. Recent case–cohort data have shown that higher circulating estradiol levels are associated with increased risk of postmenopausal ER+ breast cancer, whereas higher SHBG concentrations show an inverse association with risk [47].

SHBG concentrations are themselves influenced by metabolic and hormonal factors. Obesity and insulin resistance are commonly associated with reduced hepatic SHBG production, thereby increasing the fraction of circulating bioavailable sex steroids. In postmenopausal women with obesity, this effect may act in parallel with enhanced aromatase expression in adipose stromal cells, resulting in both increased estrogen synthesis and increased estrogen bioavailability. This combined endocrine effect may contribute to the development and progression of ER+ breast cancer and may also influence endocrine treatment response [48].

Beyond its role as a passive carrier protein, SHBG may also exert additional biological effects relevant to breast cancer. Experimental and molecular evidence suggests that SHBG can influence estrogen signaling at the cellular level and may participate in pathways involved in cell proliferation and endocrine responsiveness. However, these mechanisms remain less well established than its recognized role in regulating circulating free sex steroid concentrations and require further investigation [46].

SHBG represents an important component of the endocrine environment of breast cancer. By regulating the circulating fraction of bioavailable estrogens, particularly in postmenopausal women, SHBG may influence both breast cancer risk and the biological activity of estrogen-dependent tumors. Its interaction with obesity, insulin resistance, peripheral aromatization, and endocrine signaling further supports its potential relevance as a metabolic and hormonal biomarker in ER+ breast cancer [46,47,48].

### 2.4. Follicle-Stimulating Hormone and the Aromatase–Estrogen Axis

Follicle-stimulating hormone (FSH) represents an important physiological regulator of ovarian estrogen biosynthesis and is closely linked to aromatase activity. In the ovary, FSH acts predominantly at the level of granulosa cells, where it promotes the expression and activity of aromatase, the CYP19A1-encoded enzyme responsible for the conversion of androgen precursors into estrogens. Through this mechanism, androgens generated within the ovarian follicle are converted mainly into estradiol, thereby supporting estrogen production during the reproductive period [29,30].

The FSH–aromatase–estrogen relationship is integrated within the hypothalamic–pituitary–ovarian axis. During reproductive life, FSH supports follicular development and contributes to maintaining ovarian estrogen synthesis, while circulating estrogens exert negative feedback on hypothalamic and pituitary function. Thus, ovarian aromatase activity and estrogen production are closely coupled to gonadotropin-dependent follicular function. Estradiol is the predominant estrogen during the premenopausal period, reflecting the major contribution of ovarian steroidogenesis to systemic estrogen exposure [30].

This regulatory relationship changes substantially during the menopausal transition. Progressive depletion of the ovarian follicular reserve reduces the number of functional follicles capable of responding effectively to gonadotropin stimulation, leading to a marked decline in ovarian estrogen synthesis. The accompanying reduction in estrogen-mediated negative feedback is associated with increased circulating FSH levels. However, despite elevated FSH concentrations, ovarian estrogen production remains low because the functional capacity of the ovary is markedly diminished. Therefore, the increased FSH characteristic of menopause should be interpreted primarily as a consequence of reduced ovarian feedback rather than as an indicator of increased ovarian estrogen production [30].

As ovarian estrogen production declines, the relative contribution of extra-gonadal aromatization becomes increasingly important. In postmenopausal women, androgen precursors are converted into estrogens in peripheral tissues, particularly adipose tissue and breast tissue, where aromatase remains functionally active. Estrone consequently becomes a more prominent circulating estrogen, while local conversion between estrone and estradiol may continue to support estrogenic signaling within peripheral tissues [30,31,32]. This transition from predominantly ovarian to predominantly peripheral estrogen biosynthesis is particularly relevant to HR+ breast cancer, because local estrogen availability may continue to stimulate ER-dependent tumor growth despite the decline in ovarian function.

The FSH–aromatase relationship also helps explain the different therapeutic context of aromatase inhibition before and after menopause. In postmenopausal women, peripheral aromatization constitutes a major source of endogenous estrogen; therefore, inhibition of aromatase can produce a substantial reduction in estrogen availability and estrogen receptor-mediated signaling. In women with preserved ovarian function, however, the endocrine system retains the capacity to respond to reduced estrogen levels through hypothalamic–pituitary feedback and gonadotropin stimulation. Consequently, aromatase inhibition alone does not provide the same degree of sustained estrogen suppression as that achieved in the postmenopausal setting. This physiological difference is an important consideration when applying aromatase-targeted endocrine therapy across different reproductive stages [31,32].

Thus, FSH and aromatase are functionally interconnected within ovarian estrogen biosynthesis, but the importance of this relationship changes with age and menopausal status. Before menopause, FSH-dependent ovarian aromatase activity is a major determinant of estradiol production, whereas after menopause the decline in functional ovarian activity shifts estrogen biosynthesis toward peripheral aromatization. Understanding this transition provides important physiological context for the use of aromatase inhibitors in HR+ breast cancer.

### 2.5. Suppression of Ovarian Estrogen Synthesis Through Gonadotropin Inhibition

Ovarian estrogen synthesis in premenopausal women is tightly controlled by gonadotropins through the two-cell, two-gonadotropin model of steroidogenesis. Luteinizing hormone (LH) stimulates androgen production in theca cells, whereas FSH acts primarily on granulosa cells, where it promotes CYP19A1 expression and aromatase activity, enabling the conversion of androgen precursors into estrogens, particularly estradiol [49]. Thus, modulation of gonadotropin signaling can directly influence ovarian estrogen output. Although selective inhibition of FSH has mainly mechanistic and experimental relevance, suppression of both gonadotropins represents the clinically established strategy for reducing ovarian estrogen production in premenopausal women.

In clinical practice, ovarian function suppression is most commonly achieved through gonadotropin-releasing hormone agonists, which initially stimulate pituitary gonadotropin secretion but subsequently induce receptor desensitization and sustained suppression of both LH and FSH. The resulting reduction in ovarian stimulation markedly decreases estradiol production and creates a reversible postmenopausal-like endocrine environment [50,51]. This approach is particularly relevant in HR+ breast cancer, where reduction in estrogen availability limits ligand-dependent ER activation and thereby reduces estrogen-driven tumor cell proliferation.

Suppression of ovarian estrogen synthesis is also essential when AIs are used in premenopausal women. Aromatase inhibition alone decreases estrogen production but does not suppress ovarian function; the resulting reduction in estrogen-mediated negative feedback may increase hypothalamic–pituitary stimulation and maintain gonadotropin-driven ovarian steroidogenesis. Consequently, adequate ovarian function suppression is required to prevent compensatory ovarian estrogen production and to permit effective use of AIs in this setting [50,51].

The prognostic implications of reducing ovarian estrogen exposure have been evaluated extensively in the SOFT and TEXT trials. Long-term follow-up demonstrated that adding ovarian function suppression to endocrine therapy reduces the risk of breast cancer recurrence in selected premenopausal patients, while exemestane combined with ovarian function suppression provides greater protection against recurrence and distant recurrence than tamoxifen plus ovarian function suppression. After approximately 9 years of follow-up, the combined TEXT and SOFT analysis showed significant improvements in disease-free survival and distant recurrence-free interval with exemestane plus ovarian function suppression, although an overall survival advantage was not demonstrated across the entire study population [52].

More recent 15-year follow-up further supports the long-term prognostic relevance of intensified estrogen suppression, while also emphasizing that the magnitude of benefit is not uniform across all patients. In SOFT, the 15-year breast cancer-free interval was 78.6% with exemestane plus ovarian function suppression, 75.7% with tamoxifen plus ovarian function suppression, and 72.1% with tamoxifen alone. In the combined SOFT and TEXT analysis, exemestane plus ovarian function suppression significantly reduced distant recurrence compared with tamoxifen plus ovarian function suppression among patients with HER2-negative disease. The largest absolute survival benefits were observed in higher-risk subgroups, particularly very young women and patients with high-grade tumors [53].

However, the prognostic value of deeper estrogen suppression must be interpreted according to baseline recurrence risk and individual patient characteristics. Long-term ovarian suppression is associated with treatment-related consequences of estrogen deprivation, including vasomotor symptoms, sexual dysfunction, loss of bone mineral density, and potential adverse effects on quality of life. Therefore, the therapeutic objective is not simply to achieve the lowest possible estrogen concentration, but rather to obtain an appropriate degree of estrogen suppression in patients most likely to derive meaningful clinical benefit [50,51,53].

Inhibition of gonadotropin-driven ovarian steroidogenesis provides a mechanistic and therapeutic means of reducing estrogen exposure in premenopausal HR+ breast cancer. While selective FSH inhibition remains primarily of physiological and experimental interest, combined suppression of pituitary gonadotropin signaling is an established clinical strategy that decreases ovarian estrogen synthesis, facilitates effective aromatase inhibition, and may improve long-term outcomes in appropriately selected higher-risk patients [50,51,52,53].

### 2.6. Aromatase Inhibitors for Therapeutic Use

Hormonal therapies, including SERMs and AIs, aim to inhibit ER signaling or reduce estrogen biosynthesis, respectively, thereby providing effective therapeutic options for patients with HR+ breast cancer [54].

ER status represents an important prognostic and predictive factor in breast cancer, as ER+ tumors generally have a more favorable prognosis and are more likely to respond to endocrine therapy. By inhibiting estrogen production or disrupting ER signaling, endocrine therapies can suppress tumor cell proliferation and improve clinical outcomes [55]. Accurate assessment of hormone receptor status is therefore essential for guiding therapeutic decision-making. In particular, ER+ breast cancers are generally responsive to endocrine therapies targeting estrogen-dependent signaling pathways, although acquired resistance may develop over the course of treatment [56,57].

AIs play a central role in the treatment of ER+ breast cancer, particularly in postmenopausal women, by inhibiting the aromatase enzyme responsible for the conversion of androgens into estrogens in peripheral tissues. This suppression of estrogen biosynthesis reduces the availability of estrogen required for ER-mediated tumor cell proliferation and survival. Consequently, AIs can inhibit tumor progression and reduce the risk of disease recurrence following primary treatment, including surgery and, when indicated, chemotherapy [58].

In current medical practice, two main therapeutic strategies are used: blocking ER and reducing endogenous estrogen levels through the use of AIs.

From the first category, the most widely used antineoplastic drug is tamoxifen. It is used in the treatment of breast cancer in early and metastatic stages, in premenopausal women, as well as for breast cancer chemoprevention for women at risk. Tamoxifen is a SERM. It blocks the action of estrogens in breast tissue (and other tissues) by occupying ERs. When such a receptor is occupied by a SERM, estrogen can no longer influence cellular proliferation activity. This blockade is selective and has diverse consequences. One notable effect is the slowing of angiogenesis: not to a sufficient extent to completely stop the formation of new blood vessels within the tumor, but enough to induce apoptosis. Although it acts as an antagonist in breast tissue, at the level of the endometrium it behaves as a partial agonist, meaning that it induces estrogen-like effects and, consequently, endometrial cancers may occur [59].

Other selective ER modulators used therapeutically in breast cancer include raloxifene and toremifene [60].

The second category includes AIs, which are classified into steroidal (type I) and nonsteroidal (type II) inhibitors [61].

The first AI, aminoglutethimide (Figure 3), introduced into clinical use in the early 1970s, demonstrated therapeutic efficacy but had limited selectivity and significant toxicity. Its requirement for concomitant corticosteroid supplementation, due to suppression of adrenal steroidogenesis, particularly cortisol synthesis, further restricted its clinical application. It was subsequently withdrawn from clinical use because of its unfavorable safety profile and endocrine adverse effects [62].

Thus, the so-called second generation of AIs (fadrozole, vorozole, formestane) was developed in the 1980s and in the 1990s; however, this generation also exhibits reduced enzymatic selectivity (Figure 3) [63].

Finally, anastrozole, exemestane, and letrozole represent the third generation and are characterized by much higher enzymatic target specificity and significantly reduced adverse effects (Figure 3) [64].

All AIs are well tolerated (compared to tamoxifen), do not cause gynecological or thrombotic complications, but lead to bone demineralization and, consequently, an increased risk of fractures. The occurrence of musculoskeletal events such as arthralgia has also been observed in 30–36% of patients [65].

Compounds from the class of steroidal inhibitors (type I) (formestane, exemestane—Figure 3) are structural analogs of the natural substrates, testosterone and androstenedione, competing with them for binding to the enzyme’s active site. Their binding is strong, covalent, and irreversible, leading to permanent enzyme inactivation (so-called suicide inhibitors) [66].

Compounds from the class of nonsteroidal inhibitors (type II) (anastrozole, letrozole, vorozole, fadrozole—Figure 3) interact reversibly with the heme nucleus in the structure of cytochrome P450; therefore, sustained inhibition of aromatase activity depends on the continuous presence of the inhibitory agent [67]. Anastrozole was one of the first highly selective third-generation nonsteroidal AIs evaluated against tamoxifen, one of the most extensively used selective estrogen receptor modulators in breast cancer therapy. Two large randomized phase III trials, the North American Trial (NAT) and the Tamoxifen or Arimidex Randomized Group Efficacy and Tolerability (TARGET) trial, compared the efficacy and safety of anastrozole with tamoxifen in postmenopausal patients with advanced breast cancer. These studies demonstrated that oral administration of anastrozole (1 mg daily) provided comparable or improved clinical efficacy compared with tamoxifen (20 mg daily), while exhibiting a more favorable safety profile, including a lower incidence of thromboembolic events, vaginal bleeding, and endometrial complications. These findings contributed to the establishment of aromatase inhibition as a major endocrine therapeutic strategy in ER+ breast cancer [68].

A clinical study demonstrated that combining aromatase inhibition with estrogen receptor degradation may improve outcomes in HR+ metastatic breast cancer. The combination of anastrozole, a nonsteroidal AI, with fulvestrant, a selective estrogen receptor degrader (SERD), has been investigated as a strategy to achieve more complete estrogen signaling blockade compared with aromatase inhibition alone. In selected patient populations, this combined endocrine approach resulted in improved survival outcomes compared with anastrozole monotherapy [69].

Other studies have focused on observing and quantifying differences in the inhibitory effects of third-generation AIs on estrogen synthesis. It was found that anastrozole reduced estradiol levels by 84.9%, letrozole by 87.8%, and exemestane by 92.2%. The study included 7500 women who were randomly assigned one of the three compounds over a 5-year period as adjuvant therapy. Regarding side effect profiles, exemestane proved to be more favorable, being associated with lower levels of hypertriglyceridemia and hypercholesterolemia compared to anastrozole, as well as a lower incidence of osteoporosis [70].

Differences in efficacy and safety between third-generation AIs were investigated in the Femara versus Anastrozole Clinical Evaluation (FACE) trial, a randomized phase IIIb study initiated in 2008. The trial enrolled 4136 postmenopausal patients with HR+, node-positive early breast cancer who received adjuvant letrozole (2.5 mg/day) or anastrozole (1 mg/day) for 5 years. The final analysis showed no statistically significant difference in disease-free survival or overall survival between the two treatment groups, although letrozole demonstrated numerically higher 5-year disease-free survival rates compared with anastrozole. These findings indicate that both AIs provide comparable efficacy, with differences mainly related to their pharmacological profiles and tolerability [71].

In conclusion, studies have demonstrated the superior efficacy of AIs compared to tamoxifen, along with a reduction in adverse effects. Their effects in the treatment of breast cancer are due to estrogen deprivation through different mechanisms. Tamoxifen competes with estrogen at the estrogen receptor level, thereby interfering with estrogen-driven transcription. In contrast, inhibition of aromatase activity leads to a systemic reduction in estrogen levels and global estrogen deprivation, consequently reducing estrogen signaling. Anastrozole, letrozole, and exemestane have all demonstrated clearly superior efficacy compared to tamoxifen [72].

Commonly used AIs, including letrozole, exemestane, and anastrozole, are established components of endocrine therapy for both early-stage and advanced ER+ breast cancer. In the adjuvant setting, AIs are administered after surgery to reduce the risk of disease recurrence, with treatment typically continuing for at least five years [73]. Their superior efficacy compared with tamoxifen in postmenopausal women has established them as the preferred endocrine treatment for many patients with ER+ breast cancer [74,75].

Neoadjuvant endocrine therapy has also emerged as an important treatment strategy for postmenopausal patients with locally advanced ER+ breast cancer. In this setting, AIs are administered before surgery to reduce tumor size, thereby increasing the likelihood of breast-conserving surgery and potentially improving clinical outcomes. Their therapeutic effect is mediated by suppression of estrogen synthesis, depriving ER+ tumors of the hormonal stimulation required for growth [76].

AIs also play a central role in the management of metastatic ER+ breast cancer, particularly in postmenopausal women. Letrozole, anastrozole, and exemestane are recommended as first-line endocrine therapies for patients with locally advanced or metastatic disease and may also be used sequentially or in combination with targeted agents, depending on the clinical setting [77].

Because of their superior efficacy compared with tamoxifen in postmenopausal patients with ER+ breast cancer, AIs have become a cornerstone of endocrine therapy and contribute to improved clinical outcomes [74,75].

AIs selectively inhibit the aromatase enzyme, thereby suppressing the conversion of androgens to estrogens and reducing estrogen biosynthesis in peripheral tissues. This mechanism is particularly effective in postmenopausal women with ER+ breast cancer, in whom estrogen production occurs predominantly through peripheral aromatization and can therefore be markedly reduced by AI treatment [78]. In postmenopausal women, adipose tissue and other peripheral sites, including breast tissue, represent important sources of estrogen. By reducing estrogen availability, AIs suppress estrogen receptor-mediated tumor cell proliferation and have been shown to decrease the risk of disease recurrence, making them a key component of endocrine therapy for ER+ breast cancer [79]. However, AIs do not directly suppress ovarian estrogen production and therefore are not effective as monotherapy in premenopausal women with functioning ovaries [80]. In this population, AIs must be combined with ovarian function suppression to adequately suppress estrogen production and achieve effective endocrine treatment [81].

AI therapy is associated with a range of adverse effects that can negatively affect patients’ quality of life. Common AI-related toxicities include fatigue, vasomotor symptoms such as hot flashes, arthralgia and musculoskeletal discomfort, loss of bone mineral density, vaginal dryness, psychological symptoms including depression and anxiety, and, in some patients, cardiovascular complications [8,82]. The severity and persistence of these adverse effects vary considerably among individuals and may influence treatment adherence and persistence. Studies using validated quality-of-life assessment tools have demonstrated that AI-associated symptoms can adversely affect physical functioning, emotional well-being, and social activities [83]. Effective management of AI-related adverse effects therefore requires a comprehensive and individualized approach, including lifestyle modifications, supportive pharmacological interventions, and complementary strategies such as acupuncture and yoga when appropriate [84].

Optimal outcomes with AI therapy depend largely on sustained treatment adherence; however, adherence remains suboptimal because of treatment-related adverse effects, complex treatment regimens, and insufficient patient understanding of the benefits and importance of long-term therapy [85]. Studies have shown that a considerable proportion of patients discontinue AI treatment prematurely, which may compromise therapeutic efficacy and increase the risk of disease recurrence [86]. Several interventions may help improve treatment adherence, including comprehensive patient education and counseling, clear and open communication between patients and healthcare providers, simplified dosing regimens, regular monitoring and management of treatment-related adverse effects, and adequate social support.

Third-generation AIs offer substantial therapeutic benefits and exhibit high selectivity for aromatase compared with earlier generations of inhibitors [87]. A major distinction between steroidal and nonsteroidal AIs lies in the reversibility of their interaction with the aromatase enzyme. Steroidal AIs, such as exemestane, act as mechanism-based inhibitors and irreversibly inactivate aromatase, whereas nonsteroidal AIs, including anastrozole and letrozole, bind reversibly to the enzyme and can therefore dissociate from the aromatase active site [88]. These pharmacological differences are clinically relevant because they may influence the potency, selectivity, pharmacokinetic properties, and adverse-effect profiles of individual AIs, thereby contributing to treatment selection and tolerability [89].

### 2.7. Resistance to Aromatase Inhibitors

Resistance can arise through two major mechanisms: alterations in aromatase expression or activity that enable tumors to circumvent estrogen dependence, and dysregulation of pathways involved in ER signaling. In endocrine-resistant tumors, compensatory activation of alternative growth factor pathways can promote cellular proliferation independently of estrogen signaling. Notably, aromatase protein levels remain relatively stable in both endocrine-responsive and resistant breast cancer cells; however, aromatase activity may be enhanced in resistant cells through activation of the PI3K/Akt/IGFR (phosphoinositide 3-Kinase/protein kinase B/insulin-like growth factor receptor) signaling pathway [90].

Intrinsic resistance refers to pre-existing characteristics within a tumor that confer resistance to treatment before exposure to a therapeutic agent. These mechanisms may arise from genetic alterations affecting the majority of tumor cells, the presence of inherently non-responsive subpopulations such as cancer stem cells, or cellular mechanisms that promote drug efflux, metabolism, or inactivation [91]. For instance, genetic polymorphisms in apoptosis-related genes may impair the apoptotic response to chemotherapy and targeted therapies, thereby reducing treatment efficacy from the outset, even in the absence of prior drug exposure [92]. Acquired resistance develops after an initial response to therapy and results from genetic alterations or adaptive changes that emerge under treatment pressure. One important mechanism is target modification, in which therapy-induced mutations alter the structure or function of the therapeutic target, thereby reducing drug efficacy. In the context of AIs, such alterations may affect the drug-binding site of aromatase, decreasing the ability of AIs to effectively inhibit the enzyme and ultimately contributing to treatment resistance [93]. ESR1 mutations can lead to constitutive activation of ER signaling, allowing tumor cells to proliferate independently of estrogen availability. These mutations may also alter the receptor structure and reduce the effectiveness of endocrine therapies. In parallel, activation of bypass signaling pathways enables cancer cells to circumvent inhibited pathways and maintain proliferation despite AI treatment. Among these, the PI3K/Akt/ mechanistic target of rapamycin (mTOR) pathway plays an important role in promoting cell survival and growth. In addition, alterations within the tumor microenvironment may further support tumor persistence and progression under therapeutic pressure. ESR1 mutations are particularly relevant in HR+ breast cancer, where they represent an important mechanism of resistance to endocrine therapies that act by reducing estrogen signaling [94,95].

The PI3K/Akt/mTOR pathway is a key signaling cascade involved in the regulation of cell growth, proliferation, and survival [94]. Dysregulation of this pathway is frequently associated with therapeutic resistance in breast cancer [96]. Despite targeted treatment, aberrant activation of PI3K/Akt/mTOR signaling can occur through genetic alterations, including mutations or gene amplifications, thereby promoting tumor cell survival and continued proliferation. In particular, Akt1 mutations have been identified in treatment-resistant tumors, supporting the role of PI3K/Akt/mTOR pathway alterations in the development of acquired resistance [97]. In HR+ breast cancer, ESR1 mutations are particularly important as a mechanism of endocrine resistance. These alterations can result in constitutive activation of the ER, allowing tumor cells to maintain proliferation independently of estrogen availability [98]. Consequently, tumors harboring ESR1 mutations may continue to grow despite endocrine therapies designed to reduce estrogen production or signaling. By circumventing the suppressive effects of endocrine treatment, ESR1 mutations contribute to treatment resistance and have been associated with poorer clinical outcomes [95].

Both intrinsic and acquired resistance represent major challenges in the treatment of breast cancer. A better understanding of the molecular mechanisms underlying resistance, particularly the role of ESR1 mutations and dysregulated signaling pathways such as PI3K/Akt/mTOR, may facilitate the development of more effective therapeutic strategies. Continued research is therefore essential to identify novel therapeutic targets and optimize combination approaches capable of overcoming these resistance mechanisms, ultimately improving treatment efficacy and patient outcomes [99].

### 2.8. Genetic Susceptibility and Epigenetic Regulation of Breast Cancer Candidate Loci

Breast cancer susceptibility reflects not only coding mutations in high-penetrance genes but also common regulatory variants capable of modifying gene expression through epigenetically active genomic regions. Recent genetic association studies have provided evidence that breast cancer-associated single-nucleotide polymorphisms may localize within promoters, enhancers, and other regulatory elements characterized by tissue-specific epigenetic activity, thereby affecting transcriptional regulation rather than protein structure alone [100,101,102,103].

A recent case–control study investigating SHBG-associated polymorphisms identified an obesity-dependent association between rs10454142 in PPP1R21 and breast cancer risk. The risk-associated allele was specifically associated with disease susceptibility in women with obesity, and functional annotation showed that rs10454142 and linked variants map to adipose-specific regulatory regions characterized by epigenetic modifications of promoters and enhancers. These variants were also associated with altered transcription-factor binding and expression or splicing of several nearby genes, supporting a mechanistic link between inherited genetic variation, the epigenetic state of regulatory chromatin, and breast cancer susceptibility [100].

Genetic variation affecting the SHBG pathway provides an additional example of this interaction. Polymorphisms in genes related to circulating SHBG concentrations have been associated with breast cancer risk, suggesting that inherited variation may influence both hormonal exposure and transcriptional regulation. Several of these loci are located in functionally active genomic regions and may influence gene expression through regulatory mechanisms involving chromatin state and transcription-factor accessibility [101].

Similar observations have been reported for matrix metalloproteinase genes. Case–control studies evaluating polymorphisms in matrix metalloproteinase-1 (MMP1), MMP2, MMP3, MMP8, and MMP9 demonstrated associations between specific variants and breast cancer susceptibility. Functional bioinformatic analyses indicated that several breast cancer-associated variants are located within regulatory regions capable of influencing gene expression and splicing in mammary or adipose tissues. Moreover, the strength and direction of some of these associations were modified by obesity, suggesting that host metabolic status may interact with genetically determined regulatory architecture [102,103].

These findings support an emerging model in which breast cancer risk is influenced by the interaction between inherited genetic variation and the epigenetic configuration of regulatory regions. In this context, histone modifications at promoters and enhancers may alter chromatin accessibility and determine whether susceptibility-associated variants exert functional effects on candidate-gene transcription. This concept is consistent with genome-wide chromatin studies showing that breast cancer subtypes display distinct distributions of activating and repressive histone marks, including H3K4me3, H3K4ac, and H3K27me3, at genes involved in tumor-cell identity and malignant behavior [104,105].

Thus, genetic susceptibility and histone-mediated epigenetic regulation should not be considered independent processes. Rather, inherited variants may influence breast cancer risk partly through their localization within epigenetically regulated promoters and enhancers, while tumor-associated remodeling of histone modifications can further alter the expression of these candidate genes. This genetic–epigenetic interplay provides additional biological support for therapeutic strategies targeting chromatin-modifying enzymes in breast cancer.

## 3. Histone Deacetylases: Biological Functions, Carcinogenesis and Therapeutic Targets in Breast Cancer

HDACs are a class of enzymes predominantly localized in the nucleus that catalyze the removal of acetyl groups from ε-amino acetylated lysine residues, allowing tighter binding of histones to DNA. The coiling of the DNA helix around numerous histone molecules is important, as its expression is regulated by acetylation/deacetylation [106].

HDACs actually represent a superfamily of enzymes, divided into four classes based on sequence homology and domain organization (relative to yeast-derived enzymes), as follows:

▪ Class I, structurally homologous to the yeast Rpd3 form, includes HDAC1, 2, 3, and 8 and is localized in the nucleus. They promote cellular proliferation while simultaneously suppressing apoptotic processes.
▪ Class II, homologous to the yeast Hda1 protein, can be further divided into two subclasses: IIa (including HDAC 4, 5, 7, and 9) and IIb (including HDAC6 and 10). This class shows tissue-specific expression and is capable of shuttling between the nucleus and cytosol, suggesting that these isoforms may also participate in the deacetylation of non-histone proteins. They promote angiogenesis and enhance cancer cell migration.
▪ Class III, also known as sirtuins (SIRT1–SIRT7), comprises a family of proteins homologous to the yeast Sir2 protein. Unlike the zinc-dependent class I, II, and IV HDACs, sirtuins are NAD^+^-dependent deacetylases and are insensitive to the classical HDAC inhibitor trichostatin A (TSA). The seven mammalian sirtuins exhibit distinct subcellular localizations, including nuclear, cytoplasmic, and mitochondrial compartments, reflecting their diverse biological functions in gene regulation, metabolism, stress responses, DNA repair, and aging. They promote apoptosis and autophagy while also stimulating cellular proliferation.
▪ Class IV is represented by a single member, HDAC11, which shares structural and sequence homology with both class I and class II HDACs. Although its biological functions are less well characterized than those of other HDAC family members, HDAC11 has been implicated in the regulation of immune responses, metabolism, and tumor progression [107,108].

Among the 18 HDAC isoforms identified in humans, class I HDACs, particularly HDAC1, HDAC2, and HDAC3, and class II HDACs, especially HDAC6, have been extensively studied in breast cancer. Dysregulated expression or activity of these enzymes can contribute to the epigenetic silencing of tumor suppressor genes, alteration of ER signaling, development of endocrine resistance, and tumor progression. Therefore, selective pharmacological targeting of specific HDAC isoforms has emerged as a promising therapeutic approach, with the potential to restore endocrine sensitivity and improve the efficacy of aromatase inhibitors in HR+ breast cancer [109].

### 3.1. HDAC Biological Functions and Carcinogenesis

Histone acetylation is a dynamic post-translational modification that plays a central role in the regulation of chromatin organization and gene transcription. This process is controlled by the opposing activities of HATs, which catalyze the addition of acetyl groups to histone proteins, and HDACs, which remove these modifications [110,111]. By regulating the acetylation status of histone and non-histone proteins, HDACs influence a broad range of cellular processes, including gene expression, cell proliferation, apoptosis, autophagy, migration, and DNA damage responses (Figure 4).

Most members of HDACs superfamily contain Zn^2+^ and are therefore designated as Zn^2+^-dependent histone deacetylases. Under normal conditions, histones carry a positive charge due to amino groups present in lysine and arginine residues. These allow histones to interact readily with negatively charged phosphate groups on both strands of the DNA helix and bind to DNA. Acetylation, a normal cellular process mediated by HATs, neutralizes this positive charge by converting amines into amides, thereby reducing the ability of histones to bind DNA. This permits chromatin relaxation and, consequently, gene transcription. HDACs remove acetyl groups, allowing tighter binding of histones to DNA (by increasing ionic interactions), resulting in a more compact chromatin structure that prevents transcription. Thus, this reversible modification can be considered a major epigenetic mechanism for chromatin remodeling and gene expression control [112].

Numerous studies have examined the acetylation of lysine residues on histones H3 and H4, making these the most extensively investigated types of histone modifications. The degree of acetylation has been shown to depend on the balance between the activities of HATs and HDACs [110].

Recent studies have highlighted the synergistic effects of combinatorial histone modifications in regulating chromatin structure, providing new insights into the complexity of HDAC function [113]. In particular, combinations of histone modifications can generate distinct regulatory signals that influence chromatin organization and gene transcription. HDAC-mediated deacetylation may disrupt the balance between acetylated and methylated lysine residues, thereby modifying chromatin accessibility at transcription start sites. The existence and functional relevance of this acetyl–methyl lysine modification have been supported by recent studies [114].

Other studies have shown that HDACs regulate the expression of numerous genes through direct interactions with transcription factors such as transcription factor family (E2F), signal transducer and activator of transcription 3 (STAT3), and tumor protein p53 (p53). In addition, these enzymes participate in the deacetylation of non-histone proteins that control key cellular processes, including cell cycle progression, differentiation, and apoptosis, thereby influencing overall cellular homeostasis [115].

One classical mechanism involves the interaction between the retinoblastoma protein (Rb) and E2F transcription factors. E2F proteins regulate a broad group of genes required for DNA replication and progression through the G1/S transition. In quiescent or growth-restricted cells, hypophosphorylated Rb binds activator E2Fs and restrains their transcriptional activity. Early mechanistic studies demonstrated that Rb-dependent transcriptional repression can involve recruitment of HDAC-containing complexes to E2F-responsive promoters. Through these interactions, Rb does not merely sequester E2F but can participate in the establishment of a local transcriptionally restrictive environment. This model provides a direct mechanistic connection between classical cell-cycle regulation and epigenetic control [116]. However, more recent work has refined this concept, indicating that HDAC activity is not universally required for repression of all E2F/Rb-regulated cell-cycle genes; instead, HDACs appear to modulate or reinforce repression in a promoter- and cell-context-dependent manner [117].

The recruitment of HDAC1/HDAC2 to DNA frequently occurs through transcriptional corepressor systems rather than through direct DNA binding. Complexes containing switch-independent 3 proteins (Sin3 proteins) are among the best-characterized examples, whereas other nuclear receptor corepressors, including nuclear receptor corepressor 1 (N-CoR) and silencing mediator for retinoid and thyroid hormone receptors (SMRT), associate with HDAC-containing transcriptional regulatory networks. These scaffolding proteins permit the assembly of multiprotein complexes capable of integrating signals from transcription factors, nuclear receptors, and chromatin-associated proteins. In breast epithelial cells, this mechanism is particularly relevant because nuclear receptors such as the ER dynamically recruit both transcriptional coactivators and corepressors, allowing histone acetylation status to influence estrogen-responsive gene expression. Thus, altered composition or localization of HDAC-containing complexes may modify not only cell-cycle regulation but also hormone-dependent transcriptional programs that are central to breast cancer biology [118,119].

DNA methylation provides another major pathway through which HDAC complexes can be targeted to specific loci. Methyl-CpG-binding protein 2 (MeCP2) recognizes methylated CpG-containing DNA and can recruit transcriptional repression machinery that includes Sin3 and HDACs. This mechanism provides a molecular explanation for the functional cooperation between DNA methylation and histone deacetylation: DNA methylation generates a recognition signal, methyl-binding proteins translate this signal into protein recruitment, and associated chromatin-modifying enzymes reinforce transcriptional silencing. The original demonstration that methylated DNA and MeCP2 recruit HDAC-containing complexes established a direct causal relationship between DNA methylation and histone-dependent transcriptional repression. Although this mechanism was initially described more than two decades ago, it remains fundamental to current models of epigenetic gene silencing.

Accordingly, the transcriptional consequences of HDAC activity cannot be interpreted simply in terms of the total cellular abundance of HDAC1 or HDAC2. Appropriate targeting of these enzymes to selected promoters is equally important. If recruitment is impaired, HDAC1/HDAC2 may remain enzymatically competent but fail to deacetylate the relevant nucleosomes. The expected consequence is a local increase in histone acetylation, altered interactions with heterochromatin-associated proteins, and reduced stability of transcriptionally repressed states. This concept explains why histone hyperacetylation may occur even when global HDAC expression or activity is not completely lost. In other words, a defect in where an HDAC complex is located may be functionally as important as a defect in whether the enzyme itself remains active [120].

This distinction is particularly relevant to the interpretation of TSA experiments. TSA is a classical broad-spectrum HDAC inhibitor that blocks the catalytic activity of several zinc-dependent HDACs and has historically been used to determine whether transcriptional repression depends on histone deacetylation. In breast cancer cells, Lu et al. demonstrated that E2F1- and E2F4-containing HDAC complexes repress the tumor-suppressor gene ARHI (Aplasia Ras Homolog I; DIRAS3). Breast cancer cells displayed increased E2F1/E2F4 expression and DNA-binding activity, while treatment with TSA reduced E2F binding to the ARHI promoter and reversed E2F-mediated promoter repression. Importantly, Rb enhanced E2F1-mediated repression, supporting the existence of a functional Rb/E2F/HDAC regulatory axis in breast cancer cells [121].

These findings illustrate an important point: in cancer, E2F-HDAC complexes may not exclusively repress proliferation-promoting genes. Depending on promoter context, they can also silence tumor-suppressor genes. This duality helps explain why the effects of HDAC dysregulation are complex and why HDAC inhibition does not simply convert a repressed cell into a more proliferative state. In some genomic regions, loss of HDAC recruitment may permit inappropriate expression of cell-cycle genes; in other regions, inhibition of an HDAC-containing repressor complex may restore expression of tumor-suppressor genes. Therefore, the biological outcome depends on the repertoire of promoters occupied by HDAC1/HDAC2 and the transcription factors associated with these complexes.

More recent evidence has further challenged the traditional assumption that HDAC catalytic activity is indispensable for Rb-mediated repression of cell-cycle genes. Barrett et al. examined E2F:Rb and DREAM (DP, dimerization partner; RB-like, retinoblastoma-like proteins p107/RBL1 and p130/RBL2; E2F; and MuvB, multi-vulval class B complex) complexes in multiple arrested cell models and found that pharmacological HDAC inhibition did not generally derepress Rb/DREAM target genes. Likewise, loss of SIN3B did not cause widespread activation of cell-cycle genes, and combined SIN3A/SIN3B depletion produced only moderate effects that appeared largely independent of Rb/DREAM. The authors concluded that E2F:Rb complexes can repress many cell-cycle genes independently of HDAC enzymatic activity [117]. These contemporary data suggest that HDAC1/HDAC2 should be viewed as context-dependent modulators and stabilizers of transcriptional repression, rather than universally essential components of every Rb/E2F-repressive event.

In HR+ breast cancer, the relevance of HDAC2-containing regulatory complexes is additionally strengthened by their interaction with estrogen-dependent transcriptional networks. Forkhead Box A1 (FOXA1) is a pioneer transcription factor that controls chromatin accessibility at numerous ER-responsive genomic regions. HDAC2-mediated modulation of FOXA1 acetylation can therefore alter FOXA1 chromatin occupancy and modify hormonal responses, providing a mechanistic bridge between receptor tyrosine kinase signaling, histone deacetylation, ER-dependent transcription, and endocrine resistance [122].

From this perspective, the mechanism summarized in Figure 5 has several implications for breast cancer. Under physiological conditions, Rb/E2F complexes and associated chromatin regulators contribute to controlled transcription of cell-cycle-related genes. Recruitment of HDAC1/HDAC2-containing complexes to selected promoters can reinforce local transcriptional repression by promoting deacetylation and facilitating the maintenance of a restrictive chromatin environment. Methylated DNA and methyl-CpG-binding proteins such as MeCP2 provide an additional mechanism for concentrating these complexes at specific loci. Conversely, impaired recruitment, altered complex composition, or dysregulated HDAC activity can disturb promoter-specific histone acetylation patterns and modify gene expression [123].

Under physiological conditions, hypophosphorylated Rb binds E2F transcription factors at promoters of cell-cycle-related genes and contributes to transcriptional repression by recruiting HDAC1/HDAC2-containing multiprotein complexes. Corepressors such as mSin3, N-CoR, and SMRT, together with methyl-CpG-binding proteins such as MeCP2, facilitate the targeting of these complexes to specific genomic regions, including methylated promoters. Histone deacetylation and associated chromatin changes reinforce transcriptional repression and limit inappropriate transition from G1 phase to S phase of the cell cycle (G1/S progression). When HDAC recruitment or targeting is impaired, histone acetylation may increase locally, transcriptional repression is weakened, and selected E2F-responsive genes may become aberrantly activated, favoring dysregulated cell-cycle progression. Pharmacological HDAC inhibition by TSA further illustrates the contribution of HDAC activity to Rb-mediated transcriptional repression. In breast cancer, disruption of this regulatory network may contribute to abnormal proliferation and altered transcriptional programs.

In malignant mammary cells, these alterations may produce several apparently opposing but biologically compatible effects. Impaired targeting of HDAC complexes may reduce repression of selected E2F-responsive or proliferation-associated genes, thereby facilitating aberrant cell-cycle progression. At the same time, inappropriate recruitment or excessive HDAC activity at other genomic regions may silence tumor-suppressor genes or alter ER-dependent transcription. Thus, the oncogenic consequence is not determined simply by a global increase or decrease in histone acetylation but by redistribution and dysregulation of promoter-specific epigenetic control [124].

The therapeutic implications are equally important. HDACi can disrupt transcriptional repressor complexes and modify histone as well as non-histone acetylation, which may restore tumor-suppressor gene expression, interfere with oncogenic transcription, and sensitize cancer cells to other treatments. However, because HDAC1/HDAC2 participate in multiple and sometimes opposing transcriptional programs, therapeutic responses are likely to depend strongly on tumor subtype, genomic context, and the specific HDAC-containing complexes present in each tumor. Current research therefore increasingly focuses on isoform-selective or dual-target HDACi rather than nonspecific global HDAC inhibition [118].

Overall, Figure 5 illustrates a model in which Rb/E2F-mediated transcriptional repression is integrated with promoter-specific epigenetic regulation through HDAC1/HDAC2-containing complexes. The key concept is that transcriptional repression depends not only on HDAC catalytic activity but also on correct recruitment of these enzymes to appropriate genomic loci. Disturbance of this targeting can alter histone acetylation, weaken repression of selected genes, and contribute to abnormal transcriptional programs. In breast cancer, however, this mechanism is highly context dependent: HDAC1/HDAC2 can contribute both to repression of proliferation-associated genes and to silencing of tumor-suppressor or hormone-responsive genes. Consequently, HDAC dysregulation should be interpreted as a disturbance of gene-specific epigenetic homeostasis, rather than as a simple linear pathway from HDAC loss to increased proliferation [11].

The integration of Rb/E2F signaling with HDAC1/HDAC2-containing corepressor complexes represents an important epigenetic mechanism for controlling cell-cycle-related gene expression, as illustrated in Figure 5.

HDACs also play a role in gene expression regulation mediated by nuclear receptors. ERs, members of a nuclear receptor superfamily, regulate genes essential for mammary and ovarian functions. HDAC1 interacts with ER, leading to suppression of its transcriptional activity. This interaction, which is reduced in the presence of estrogens, is mediated through the activation function-2 (AF-2) domain on DNA [125]. Other studies have highlighted the involvement of a multiprotein complex composed of HDAC, DNA methyltransferase (DNMT), and the Rb [126]. Analyses of normal tissues and a panel of human cancer cell lines, including the breast cancer cell lines MCF-7 and MDA-MB-231, demonstrated a loss of monoacetylated and trimethylated histone H4, predominantly involving acetylation at lysine 16 (H4K16ac) and trimethylation at lysine 20 (H4K20me3) [127].

In conclusion, several mechanisms have been proposed to explain how histone deacetylases contribute to cancer development. To date, most research has focused on the aberrant recruitment of HDACs to specific gene promoters through interactions with fusion proteins, which are associated with the chromosomal translocations characteristic of breast malignant cells [128].

However, the role of HDACs in cancer extends beyond histone deacetylation to include the deacetylation of non-histone proteins. In particular, HDAC-dependent deacetylation can regulate the stability and transcriptional activity of the tumor suppressor p53, thereby influencing cell-cycle arrest and apoptosis [129].

Another study identified a mutation in HDAC2 that resulted in the loss of its expression and enzymatic activity. While the precise molecular consequences of this alteration remain unclear, the authors suggest that it could lead to the activation of oncogenes. Supporting this, evidence indicates that mutations or functional loss of class I histone deacetylases may contribute to cancer development. Specifically, the tumor suppressor gene *Rb* relies on the recruitment of class I HDACs to repress target gene transcription. When HDAC activity is lost due to mutation, it is proposed that genes normally regulated by *Rb* may become aberrantly expressed, thereby compromising their tumor-suppressive function [130].

Sirtuins, or class III HDACs, have attracted considerable attention in breast cancer because of their diverse roles in the regulation of gene expression, cellular stress responses, DNA repair, cell-cycle progression, apoptosis, and genomic stability. Among the seven mammalian sirtuins, SIRT1 is one of the best-characterized members and regulates the acetylation status of both histone and non-histone proteins. SIRT1 deacetylates histones, particularly at H4K16 and H3K9, as well as several transcriptional and DNA-repair regulators, including p53, the histone acetyltransferase p300, and the DNA repair protein Ku70, thereby influencing transcriptional activity, DNA damage responses, cell-cycle control, and apoptosis [131]. In breast cancer, dysregulation of SIRT1 has been associated with alterations in histone acetylation and the expression of genes involved in tumor suppression and cancer progression. Importantly, inhibition of SIRT1 has been shown to increase H4K16ac and H3K9ac levels and to promote the re-expression of tumor suppressor genes in breast cancer cell models, supporting a potential role for aberrant SIRT1-dependent deacetylation in breast cancer development and progression [132,133].

HDACs interact with several critical signaling pathways, including those regulated by nuclear factor-κB (NF-κB), p53, and heat-shock protein 90 (HSP90), thereby influencing cellular responses to stress, survival, and proliferation. In cancer, where the NF-κB pathway is frequently constitutively activated, HDAC-mediated deacetylation of pathway components can enhance pro-survival and proliferative signaling, contributing to tumor progression [134,135]. Similarly, HDACs can negatively regulate the tumor-suppressive activity of p53. Deacetylation of p53 reduces its transcriptional activity and may consequently impair the induction of apoptotic responses, allowing cancer cells to evade programmed cell death [136]. A specific example of this interplay during cancer progression is the interaction between HDAC5 and special AT-rich sequence-binding protein 1 (SATB1), a chromatin-organizing factor involved in the regulation of tumor-suppressor gene expression [137]. The dysregulated expression and activity of HDACs across different malignancies highlight their dual role in cancer biology, where they can contribute to oncogenic signaling while simultaneously representing attractive therapeutic targets. A deeper understanding of the complex interactions between HDACs, key regulatory proteins, and associated signaling pathways may facilitate the development of more effective anticancer strategies. In particular, combining HDACi with conventional therapeutic approaches represents a promising strategy for overcoming treatment resistance, enhancing therapeutic efficacy, and ultimately improving patient outcomes [138].

In summary, different HDAC subtypes contribute to a complex mechanism–phenotype regulatory network through chromatin remodeling, modification of non-histone proteins, and modulation of key signaling pathways, including NF-κB and p53. Aberrant activation of class I HDACs, particularly HDAC1 and HDAC3, can promote tumor cell proliferation through the epigenetic repression of tumor-suppressor genes. Class II HDACs, including HDAC5 and HDAC7, have been implicated in tumor progression and metastasis by regulating processes such as epithelial–mesenchymal transition (EMT) and angiogenesis. In contrast, HDAC11, the sole member of class IV, may exert context-dependent and potentially opposing effects depending on the tumor type and microenvironment. Together, these mechanisms provide an important framework for understanding the functional diversity of HDACs and their distinct contributions to tumor development and progression.

HDACs have emerged as important therapeutic targets for the development of potent anticancer agents [139]. Their contribution to tumor growth may be mediated, at least in part, through post-translational regulation of key angiogenic factors, including hypoxia-inducible factor-1α (HIF-1α) and vascular endothelial growth factor (VEGF) [140]. Different HDAC isoforms are involved in the regulation of cell-cycle progression, cellular differentiation, and tumor growth. Accordingly, HDAC inhibition has been shown to suppress angiogenesis and induce cell-cycle arrest, mitotic cell death, and autophagic cell death [141]. In addition, HDACi can promote apoptosis and, in some experimental settings, exhibit preferential cytotoxicity toward cancer cells. The involvement of HDACs in tumor development is further supported by their aberrant expression in several cancer types. In breast cancer, HDAC1, HDAC2, and HDAC3 are frequently expressed at high levels, whereas increased expression of HDAC2 and HDAC3 is particularly associated with aggressive clinicopathological features and tumor progression [125,142].

Importantly, the rationale for targeting HDACs in breast cancer is supported not only by their general role in chromatin regulation, but also by evidence of altered HDAC expression during tumor development. In human breast cancer, class I HDACs, particularly HDAC1, HDAC2, and HDAC3, show differential and frequently increased expression compared with non-neoplastic tissue. Increased HDAC2 and HDAC3 expression has been associated with clinicopathological features of disease progression, while HDAC1 has been implicated in breast cancer cell proliferation and migration [119,142]. These findings indicate that dysregulated HDAC expression is not merely an epiphenomenon of malignant transformation but may contribute functionally to tumor growth and progression.

This biological involvement provides a rationale for considering HDACs as therapeutically exploitable epigenetic regulators. HDACs influence multiple processes relevant to breast cancer biology, including transcriptional repression, cell-cycle progression, apoptosis, differentiation, angiogenic signaling, and the regulation of non-histone proteins. Accordingly, pharmacological HDAC inhibition can induce cell-cycle arrest and apoptosis and can interfere with signaling networks that sustain tumor-cell survival [139,140,141]. In ER+ breast cancer, this therapeutic rationale is particularly relevant because HDACs participate in the regulation of ER-associated transcription and can influence the expression of genes involved in hormone-dependent proliferation and endocrine responsiveness.

However, current evidence also indicates that HDAC dependency is heterogeneous and should not be regarded as a universal vulnerability of all ER+ tumors. The biological effects of HDAC inhibition depend on the HDAC isoform involved, the transcriptional context, and the molecular characteristics of the tumor. Thus, HDACs are best considered context-dependent therapeutic vulnerabilities whose clinical exploitation may require greater isoform selectivity and improved biomarker-based patient stratification [139,140].

### 3.2. Molecular Mechanisms of Action of Histone Deacetylase Inhibitors

Studies have also demonstrated that HDACs regulate the acetylation and function of numerous non-histone proteins. More than 50 non-histone proteins have been identified as substrates of HDACs, including transcription factors such as runt-related transcription factor 3 (RUNX3), p53, and cellular Myc (c-Myc), nuclear hormone receptors such as ERα and androgen receptor (AR), and signaling molecules such as SMAD family member 7 (Smad7) [143]. In breast cancer cell lines, including ERα-positive MCF-7 and ERα-negative MDA-MB-231 cells, HDACi have been shown to induce the expression of the cell-cycle regulator p21^WAF1/CIP1 (cyclin-dependent kinase inhibitor 1, CDKN1A), leading to inhibition of cyclin/ cyclin-dependent kinase (CDK) activity and subsequent cell-cycle arrest [144]. Furthermore, HDAC inhibition can increase p53 acetylation and stability, thereby enhancing its transcriptional activity toward p53-responsive genes, including *p21*. In breast cancer cells, increased p53 acetylation following HDAC inhibition has been associated with upregulation of p21, cell-cycle arrest, and induction of apoptosis [145].

Furthermore, HDACi exert pleiotropic effects in breast cancer cells, including modulation of angiogenic signaling, induction of intracellular reactive oxygen species (ROS), and disruption of chaperone protein function. HDAC inhibition has been reported to interfere with the VEGF/vascular endothelial growth factor receptor 2 (VEGFR-2) angiogenic pathway and to reduce VEGFR-2 signaling [146]. In addition, HDACi can increase intracellular ROS levels, contributing to oxidative stress, DNA damage, and apoptosis in breast cancer cells. HDACi also affect the function of the molecular chaperone HSP90 by increasing its acetylation. In its hypoacetylated state, HSP90 stabilizes a variety of client proteins, including BCR::ABL1 fusion protein (BCR-ABL), epidermal growth factor receptor (EGFR), and Erb-B2 receptor tyrosine kinase 2 (ErbB2)/HER2, thereby protecting them from degradation. HDAC inhibitor-induced hyperacetylation of HSP90 impairs its chaperone activity and promotes the degradation of these client proteins [147].

Epigenetic regulation represents a critical interface between chromatin organization, transcriptional control, and cell-cycle progression. In mammary epithelial cells, DNMTs, HDACs, and histone methyltransferases (HMTs) cooperate to establish and maintain appropriate patterns of gene expression. Rather than functioning independently, these enzymes form interconnected regulatory networks that determine whether particular genomic regions remain transcriptionally inactive or become accessible to transcription factors. Alterations affecting these mechanisms are increasingly recognized as important contributors to breast cancer development, progression, phenotypic plasticity, and therapeutic resistance. Experimental studies have demonstrated that simultaneous perturbation of DNA methylation and histone deacetylation profoundly modifies breast cancer cell proliferation, migration, invasion, and immune-related transcriptional programs, emphasizing the functional interdependence of these epigenetic systems [148].

As illustrated in Figure 6, under normal conditions, DNMT3A, HDACs, and HMTs contribute to maintaining the transcriptional repression of genes whose inappropriate expression could stimulate cell-cycle progression. DNMT3A catalyzes de novo DNA methylation, particularly at cytosine–phosphate–guanine (CpG)-rich regulatory regions, thereby contributing to the establishment of long-term transcriptional programs. HMTs add methyl groups to specific lysine or arginine residues of histones, and the biological consequences depend on the residue involved. HDACs, including the class I enzyme HDAC2, remove acetyl groups from histone and non-histone proteins and therefore participate in the dynamic control of transcription. In cancer cells, disruption of the balance among DNA methylation, histone methylation, and histone acetylation may lead to inappropriate silencing of tumor-suppressor genes while simultaneously permitting activation of proliferative and survival pathways. Recent experimental evidence provides direct support for the oncogenic relevance of DNMT3A in breast cancer: DNMT3A-mediated methylation of the ADAM metallopeptidase with thrombospondin type 1 motif 8 (ADAMTS8) promoter was shown to suppress ADAMTS8 expression and enhance EGFR–mitogen-activated protein kinase kinase–extracellular signal-regulated kinase (ERK) signaling, promoting breast cancer cell proliferation, migration, and invasion [148,149].

One of the most important downstream systems connecting epigenetic regulation with cellular proliferation is the Rb/E2F pathway. Rb functions as a major gatekeeper of the G1/S transition [150]. In its active, hypophosphorylated state, Rb binds E2F transcription factors and limits their ability to activate genes required for DNA replication and S-phase entry. Consequently, E2F-responsive genes involved in nucleotide synthesis, DNA replication, replication licensing, and cell-cycle progression remain transcriptionally restricted. When mitogenic stimulation activates cyclin D–CDK4/6 complexes, Rb becomes progressively phosphorylated and loses its capacity to restrain E2F. E2F is then released and drives the transcriptional program required for progression from G1 into S phase. This pathway is particularly relevant to HR+ breast cancer, where ER and growth-factor signaling converge on cyclin D–CDK4/6–Rb signaling [151,152].

The relationship between epigenetic mechanisms and the Rb/E2F pathway extends beyond simple regulation of chromatin accessibility. Rb-dependent transcriptional repression involves recruitment of chromatin-modifying complexes to E2F-regulated promoters, allowing cell-cycle control to be reinforced epigenetically. Therefore, disruption of DNA methylation or histone-modification patterns may reduce the stability of transcriptionally repressed states and facilitate inappropriate activation of E2F-responsive genes. In cancer cells, this effect can cooperate with classical mechanisms of Rb inactivation, such as excessive cyclin D–CDK4/6 signaling, Rb phosphorylation, RB transcriptional corepressor 1 (RB1) loss, or alterations affecting cyclin E/CDK2 signaling. Breast cancer models with acquired resistance to CDK4/6 inhibitors have provided particularly convincing evidence for the importance of this mechanism. Kim et al. demonstrated that resistance can develop through sequential activation of E2F involving Rb protein degradation followed by c-Myc-mediated amplification of E2F transcriptional activity [151]. Thus, sustained E2F activation can represent a final common pathway through which multiple oncogenic and epigenetic alterations promote cell-cycle escape.

HDAC2 represents an especially relevant component of this regulatory network. Although the simplified model in Figure 6 illustrates how disruption of HDAC2-mediated control may alter histone acetylation and subsequently disturb gene expression, the role of HDAC2 in breast cancer is not unidirectional. HDAC2 does not function simply as a tumor suppressor whose loss inevitably increases proliferation; rather, abnormal HDAC2 expression, recruitment, or enzymatic activity can generate different biological effects depending on the molecular context. This distinction is important because contemporary studies increasingly associate increased HDAC2 activity with malignant phenotypes in selected breast cancer subtypes.

The consequences of disrupted epigenetic regulation are especially important in HR+ breast cancer. ER signaling stimulates transcription of genes that promote proliferation, including genes converging on cyclin D–CDK4/6 activity. Once CDK4/6 phosphorylates Rb, the Rb–E2F complex dissociates, allowing E2F-dependent transcription and progression through the G1/S checkpoint. Breast cancer cells can acquire resistance by restoring E2F activity through alternative routes, even when CDK4/6 remains pharmacologically inhibited. Experimental models have demonstrated Rb degradation, activation of c-Myc, cyclin E/CDK2 signaling, and activation of alternative receptor tyrosine kinase pathways as mechanisms capable of restoring proliferative transcription [151,152,153].

Epigenetic remodeling may facilitate this adaptation by changing the accessibility and transcriptional responsiveness of genes involved in estrogen signaling and cell-cycle progression. A particularly relevant recent example is the interaction between HDAC2 and the pioneer transcription factor FOXA1. FOXA1 is essential for defining chromatin accessibility at many ER-regulated genomic sites and therefore plays a central role in luminal breast cancer biology. Recent experimental evidence suggests that HDAC2-dependent deacetylation of FOXA1 can alter its chromatin binding and influence ER signaling and hormone responsiveness. This provides a direct mechanistic connection between HDAC2 activity, chromatin organization, and endocrine therapy response [154]. Such findings indicate that HDAC2 dysregulation may affect breast cancer progression not only through global changes in histone acetylation but also by modifying key transcription factors that regulate estrogen-dependent gene expression.

The therapeutic relevance of this interaction is supported by studies investigating HDAC inhibition in endocrine-resistant breast cancer. Entinostat is a class I HDAC inhibitor that targets HDAC1 and HDAC3 and also affects HDAC2-associated epigenetic programs. In a randomized phase III trial conducted in Chinese patients with HR+ advanced breast cancer previously exposed to endocrine therapy, the combination of entinostat and exemestane increased median progression-free survival from 3.72 months with placebo plus exemestane to 6.32 months with entinostat plus exemestane [155]. Although results obtained with HDACi have not been uniformly positive across all clinical trials and populations, these findings provide clinical proof-of-concept that modification of HDAC-dependent epigenetic programs can influence endocrine responsiveness in selected patients.

Importantly, E2F itself should no longer be considered exclusively a passive downstream target of Rb. Recent studies suggest that E2F1 can participate actively in oncogenic signaling networks. In breast cancer cells, activation of an E2F1/ SH3 domain-binding kinase 1 (SBK1) regulatory axis was recently shown to stimulate Notch signaling and promote malignant progression [156]. This observation supports the concept that persistent E2F activation following impairment of Rb-mediated repression can initiate secondary transcriptional programs that promote proliferation and tumor progression beyond the immediate effects on G1/S transition.

Taken together, the pathway represented in Figure 6 illustrates the convergence of epigenetic and cell-cycle regulatory mechanisms in breast cancer. Under physiological conditions, DNMT3A, HDACs, including HDAC2, and HMTs contribute to maintaining appropriate transcriptional repression, while active hypophosphorylated Rb restrains E2F-dependent transcription and prevents inappropriate entry into S phase. In breast cancer, disruption of this equilibrium through abnormal DNA methylation, altered histone acetylation or methylation, aberrant HDAC2 expression or recruitment, excessive CDK4/6 activity, or functional impairment of Rb can facilitate inappropriate activation of E2F-dependent genes. The resulting transcriptional reprogramming promotes sustained proliferation and can also influence DNA repair, tumor-cell plasticity, endocrine resistance, and response to chemotherapy [149,151,152].

Therefore, the most biologically appropriate interpretation of Figure 6 is not that loss of HDAC2 alone directly causes breast cancer, but rather that HDAC2 dysregulation contributes to a broader disturbance of epigenetic homeostasis that can impair Rb/E2F-dependent control of proliferation [155].

The interplay between epigenetic regulators and the Rb/E2F axis provides an important mechanistic link between chromatin remodeling and cell-cycle control, as summarized in Figure 6.

Other studies have shown that a possible mediator of apoptosis induced by certain specific histone deacetylase inhibitors (romidepsin = FK228 and m-carboxycinnamic acid bis-hydroxamide = CBHA) is Bcl-2 modifying factor (Bmf). Overexpression of HDAC1 reduces Bmf expression. FK228 induces acetylation of histones H3 and H4 in the Bmf promoter region, suggesting that hyperacetylation leads to transcriptional activation of Bmf. Silencing Bmf transcription protects cells from FK228- or CBHA- induced cell death, mitochondrial membrane potential disruption, and DNA fragmentation [157].

Another mechanism underlying the anticancer effects of HDACi is the regulation of cellular differentiation through activation of protein kinases (such as ERK). Protein kinases generally modulate biological processes such as cell growth, differentiation, and apoptosis. Studies have demonstrated that HDACi increase the binding and transactivation activity of the transcription factor AP-1 via ERK activation, increase the expression of c-Jun (a binding protein), and induce its phosphorylation in certain tumor cells. Although the exact mechanism by which HDACi affect ERK remains unclear, it is assumed that they may induce the synthesis of an as-yet unidentified factor that activates the ERK signaling pathway, or that they are incorporated into phospholipid molecules that activate ERK via phosphatidylinositol 3-kinase [10,158].

Certain HDAC inhibitors downregulate the expression of the enzyme DNMT1 in the nucleus of breast tumor cells. Co-treatment with the proteasome inhibitor MG-132 abolishes the ability of panobinostat (LBH589) to reduce DNMT1 activity, suggesting that DNMT1 degradation is mediated via the proteasomal pathway. Deletion of 120 amino acid residues from the N-terminal region of DNMT1 reduces LBH589-induced ubiquitination, indicating that this domain is essential for its proteasomal degradation. DNMT1 recruits the heat shock chaperone protein Hsp90 and forms a chaperone complex. Treatment with LBH589 induces hyperacetylation of Hsp90, thereby inhibiting this association and promoting DNMT1 ubiquitination. It is therefore concluded that DNMT1 stability is, at least in part, maintained through its association with Hsp90. Disruption of Hsp90 function by HDAC inhibitors thus represents a mechanism contributing to DNMT1 degradation via the ubiquitin–proteasome pathway [148,159].

Another relatively recent study suggests that selective inhibition of certain HDAC isoforms (such as HDAC6) may sensitize tumor cells to anticancer agents by inducing damaging DNA modifications [160].

Other possible mechanisms by which HDACi induce cell death, as well as their role in combination anticancer therapies, are still under investigation.

Ultimately, maintaining a balance between the opposing actions of histone HATs and HDACs is essential for the epigenetic regulation of gene expression. In breast cancer, disruption of this balance has been associated with aberrant histone acetylation and epigenetic reprogramming, which can alter the expression of genes involved in proliferation, differentiation, apoptosis, and tumor progression. By targeting histones and multiple non-histone proteins, HDACi can modulate cellular acetylation profiles and reverse, at least in part, aberrant epigenetic and protein functions involved in breast cancer development [10,161,162].

### 3.3. Histone Deacetylases Inhibitors for Therapeutic Use

Following the identification of the role of histone deacetylases in cancer, HDACis were developed as potential anticancer agents. The clinical relevance of this therapeutic approach was established with the FDA approval of vorinostat in 2006. HDACi can exert cytostatic and cytotoxic effects by inhibiting tumor cell proliferation and, depending on the cellular context, inducing cell-cycle arrest, differentiation, and/or apoptosis both in vitro and in vivo [163].

Structurally, HDACi are categorized into four groups: hydroxamates, cyclic peptides, aliphatic acids, and benzamides [164]. The most important representatives are summarized in Table 2.

Among the hydroxamate-class HDACi, vorinostat (SAHA) was the first to receive FDA approval in 2006 for the treatment of cutaneous T-cell lymphoma. TSA, another hydroxamate HDACi, has been extensively used as a research tool but has not been approved for clinical use. Cyclic peptides, which represent some of the most structurally complex HDACi, include romidepsin (formerly known as depsipeptide) and apicidin (Figure 7) [163].

Depsipeptide is the most important member of this class and was approved by the FDA in November 2009 (FDA, Office of Oncology Drug Products). It is a prodrug that is intracellularly converted into a reduced form containing a sulfhydryl functional group, capable of interacting with zinc in the active site pocket of class I HDACs [173].

Aliphatic acids, including butyrate, phenylbutyrate, and valproic acid, are relatively weak HDACi, requiring millimolar concentrations for activity, and are thus not regarded as first-line therapeutic agents [164].

The benzamide class of HDACi is particularly noteworthy because these compounds exhibit preferential activity against class I HDACs, which are closely associated with tumor growth and metastatic progression [174]. Tucidinostat, a benzamide-type HDACi, has not received approval from the FDA but has been approved by Chinese regulatory authorities for the treatment of advanced breast cancer [169]. In contrast, entinostat has received FDA Breakthrough Therapy designation for the treatment of advanced breast cancer, reflecting its potential therapeutic value in this setting [170].

The mechanisms of action are thought to involve changes in gene expression and modifications of non-histone proteins, mediated through epigenetic and post-translational regulation [175].

Currently, clinical studies are underway involving multiple HDACi as single agents or in combination with conventional chemotherapies or targeted therapies. This approach is pursued because resistance to these inhibitors and their limited efficacy in treating solid tumors have frequently been observed. Their combination with standard chemotherapeutic agents has shown promising enhancement of antitumor effects in both preclinical and clinical studies [164]. For example, preclinical studies have investigated combinations of HDACi with cisplatin and carboplatin (a less nephrotoxic and neurotoxic analog) [176]. Based on synergistic and additive antitumor effects, such combinations aim to maximize efficacy, reduce toxicity, and delay resistance by allowing the use of lower doses.

Resistance to HDACi has often been observed, although the molecular basis is not yet fully elucidated. Possible mechanisms involve Bcl-2, thioredoxin, peroxiredoxins, and mutations that alter tumor cell responses to therapeutic agents. Additionally, HDACi have shown limited therapeutic efficacy in solid tumors, which supports the recommendation of combining them with other therapeutic agents or immunotherapies [177]. Synergistic or additive effects have also been demonstrated in vitro and in vivo experiments when HDACi are combined with anticancer drugs (doxorubicin, 5-fluorouracil, cyclophosphamide) and/or radiotherapy [178,179].

Compared to monotherapy, studies have shown that combined treatment with HDACi and topoisomerase II inhibitors leads to increased nuclear accumulation of topoisomerase inhibitors, resulting in enhanced DNA damage, inhibition of cell growth, and ultimately cell death. A safe and feasible combination shown to be effective is that of valproic acid with epirubicin, with a 22% increase in response rate [179,180].

As previously discussed, tyrosine kinase receptors regulate important biological functions, including cell growth, organ morphogenesis, neurovascularization, regeneration, and tissue repair. These functions are dysregulated in cancer. Therefore, phase I clinical studies have investigated combined therapies involving HDACi and kinase inhibitors such as sorafenib and gefitinib in advanced or metastatic solid tumors, showing increased efficacy due to synergistic effects [158].

Another therapeutic strategy involves targeting the ubiquitin–proteasome system, which plays an essential role in the degradation of intracellular proteins and the maintenance of cellular homeostasis. Because malignant cells exhibit high rates of protein synthesis and turnover, disruption of proteasomal activity can increase cellular stress and promote apoptosis. Proteasome inhibitors, such as bortezomib, have therefore been investigated in breast cancer, particularly in combination with other anticancer agents. Recent preclinical evidence indicates that combination strategies involving bortezomib can enhance antitumor activity in breast cancer models, although the clinical efficacy of proteasome inhibition in solid tumors remains limited compared with hematological malignancies [181,182]. HDACi may provide an additional strategy for exploiting proteotoxic and cellular stress pathways, and the combination of HDAC and proteasome inhibition has shown synergistic effects in hematological malignancies; however, further investigation is required to establish whether this approach can be effectively translated to breast cancer [183].

A commonly used and effective combination is that of HDACi with hormonal therapy. For example, the vorinostat–tamoxifen combination has shown promising results in treating metastatic breast cancer. Another study addressing resistance to hormonal therapy in ER+ breast tumors demonstrated that combined treatment with vorinostat and tamoxifen resulted in tumor regression and disease stabilization in 40% of patients (ClinicalTrials.gov identifier: NCT00365599) [184].

Combination therapies involving HDACi and DNA hypomethylating agents have attracted increasing attention, as aberrant DNA methylation represents an important epigenetic mechanism involved in malignant transformation [148].

Finally, as with other therapeutic agents—especially anticancer drugs—adverse effects of HDACi must be considered. Adverse effects include thrombocytopenia, neutropenia, nausea, vomiting, diarrhea, and fatigue, with the most serious being cardiac toxicity, such as ventricular arrhythmias. In combination therapies, it is important to determine whether toxicity is attributable solely to the HDAC inhibitor or whether it is exacerbated by co-administration. Optimally, clinical trials should use biomarkers to select patients most likely to benefit from HDAC inhibitor therapy, thereby reducing adverse effects in non-responders [185,186].

HDACs and HDACi have demonstrated considerable potential as therapeutic targets in cancer by regulating multiple processes involved in tumor development and progression. However, several challenges continue to limit their clinical application. First, the efficacy of HDACi as monotherapies in solid tumors remains relatively limited, potentially due to factors such as tumor heterogeneity, hypoxic conditions within the tumor microenvironment, and inadequate drug penetration. In addition, most currently available HDACi are broad-spectrum agents with limited isoform selectivity, which may increase the risk of off-target effects and treatment-related toxicities, including hematological and cardiac adverse events [187]. Furthermore, reliable predictive biomarkers for the efficacy of HDACi have yet to be clearly established, making accurate patient selection and stratification challenging. Overall, HDACs and their inhibitors represent promising targets for epigenetic cancer therapy; however, their further clinical development requires overcoming the limitations of their efficacy in solid tumors, improving isoform selectivity, and identifying robust HDAC-related biomarkers to enable more precise patient selection and guide individualized treatment strategies [107]. To address the challenges associated with the clinical translation of HDACi, current research is increasingly focused on three major strategies: structural optimization of HDACi, targeted drug delivery, and the development of rational combination therapies. These approaches aim to improve isoform selectivity and antitumor efficacy while minimizing treatment-related toxicity. In addition, combination therapies and precision medicine approaches based on tumor molecular subtypes represent promising directions for future research, with the goal of identifying patient-specific therapeutic strategies and improving the effectiveness of HDAC-targeted treatment [188].

## 4. Therapeutic Interplay Between Aromatase Inhibition and Histone Deacetylase Targeting in Breast Cancer

Identification and validation of molecular targets involved in the regulation of cellular proliferation and gene expression represent a central direction in the development of modern anticancer therapies [189]. Aromatase (CYP19A1), a key enzyme in estrogen biosynthesis, catalyzes the conversion of androgens into estrogens and contributes to maintaining a tumor microenvironment favorable to hormone-dependent neoplasms, particularly breast cancer. Aromatase inhibition is a clinically validated therapeutic strategy; however, the development of resistance remains a major challenge [64].

In parallel, HDACs are essential epigenetic regulators that modulate chromatin structure and gene expression through the deacetylation of histone and non-histone proteins. Dysregulation of HDAC activity is implicated in tumorigenesis and in the development of therapeutic resistance, being associated with altered expression of genes involved in proliferation, differentiation, and apoptosis [190]. HDACi have demonstrated significant antitumor effects and are extensively investigated in combination therapies to improve clinical response [11].

Considering the interplay between hormonal signaling and epigenetic mechanisms, combined strategies targeting both aromatase and HDACs have attracted considerable interest. Recent studies highlight synergistic effects of these approaches, including enhanced tumor cell sensitivity and modulation of the tumor microenvironment [191]. There is a functional and indirect, yet important, relationship between aromatase and HDACs in cancer. This is not a direct enzyme–enzyme interaction, but rather one that occurs through the regulation of gene expression and cellular signaling pathways [140,192].

Therefore, investigating these enzymes as therapeutic targets offers promising perspectives for the development of more effective anticancer agents and for overcoming treatment resistance.

### 4.1. HDAC-Mediated Epigenetic Regulation of Aromatase

Aromatase, encoded by the CYP19A1 gene, is the key enzyme responsible for the final step of estrogen biosynthesis and plays a central role in the pathogenesis of HR+ breast cancer. While aromatase expression is tightly regulated under physiological conditions, its transcription is frequently dysregulated in breast tumors, resulting in increased local estrogen production within the tumor microenvironment. Unlike ovarian estrogen synthesis, which is primarily controlled by gonadotropins, aromatase expression in breast tissue is regulated through a complex network of tissue-specific promoters, transcription factors, cytokines, growth factors, and epigenetic mechanisms [17,193].

Epigenetic regulation has emerged as a critical determinant of CYP19A1 expression. Chromatin remodeling, DNA methylation, and post-translational histone modifications collectively influence the accessibility of the CYP19A1 promoter to the transcriptional machinery. Among these mechanisms, reversible histone acetylation plays a particularly important role in controlling aromatase transcription. HATs promote chromatin relaxation and transcriptional activation, whereas HDACs remove acetyl groups from lysine residues of histone tails, resulting in chromatin condensation and transcriptional repression. The dynamic balance between these opposing enzymatic activities determines the transcriptional status of numerous genes involved in breast cancer progression, including CYP19A1 [17,194].

Increasing evidence indicates that HDACs regulate aromatase expression both directly and indirectly. HDAC-mediated chromatin remodeling influences promoter accessibility, while deacetylation of non-histone proteins, including transcription factors and transcriptional co-regulators, further modulates CYP19A1 transcription. In breast cancer-associated fibroblasts and adipose stromal cells, inflammatory mediators such as prostaglandin E_2_, interleukin-6, and tumor necrosis factor-α (TNF-α) activate signaling pathways that promote a switch from physiological to tumor-associated CYP19A1 promoters, leading to enhanced aromatase expression and increased intratumoral estrogen synthesis. Epigenetic mechanisms are thought to facilitate this promoter switching by altering chromatin accessibility and transcription factor recruitment [17,193].

HDACs also interact closely with ER signaling. Beyond regulating chromatin structure, several HDAC isoforms modulate the activity of ER-associated transcriptional complexes through interactions with transcriptional co-repressors and co-activators. Consequently, aberrant HDAC activity may simultaneously influence estrogen biosynthesis through aromatase regulation and estrogen responsiveness through alterations in ER-dependent gene expression. This dual regulatory function establishes an important molecular link between endocrine signaling and epigenetic control in breast cancer [11,17].

The functional relationship between aromatase and HDACs provides a strong biological rationale for combining AIs with HDACi. HDAC inhibition may restore a more permissive chromatin state, reactivate genes involved in endocrine responsiveness, and enhance the antitumor activity of AIs. These findings have stimulated considerable interest in epigenetic combination therapies designed to overcome endocrine resistance and improve clinical outcomes in patients with HR+ breast cancer [195,196].

HDACs contribute to the epigenetic regulation of CYP19A1 by modulating chromatin accessibility and the activity of transcriptional regulatory complexes. Class I HDACs, particularly HDAC1, HDAC2, and HDAC3, have received considerable attention because of their central roles in transcriptional repression and breast cancer progression.

HDAC1 and HDAC2 are components of several transcriptional co-repressor complexes, including Sin3, NuRD (nucleosome remodeling and deacetylase), and CoREST (co-repressor for element-1-silencing transcription factor). By removing acetyl groups from histones H3 and H4, these enzymes promote chromatin condensation and reduce transcriptional accessibility. Increased expression of HDAC1 has been associated with poor prognosis, enhanced cellular proliferation, and altered expression of estrogen-responsive genes in breast cancer. Although HDAC1 does not directly regulate aromatase enzymatic activity, it influences CYP19A1 transcription by modifying chromatin organization and regulating the recruitment of transcription factors to aromatase promoters [194].

Among class I enzymes, HDAC3 appears to play a particularly important role in endocrine resistance. HDAC3 regulates several transcriptional programs involved in ER signaling, cell-cycle progression, and adaptive responses to estrogen deprivation. Experimental studies suggest that HDAC3-mediated chromatin remodeling contributes to the transcriptional plasticity observed in breast cancer cells exposed to long-term aromatase inhibitor therapy, thereby facilitating resistance to endocrine treatment [11,17].

In contrast to the zinc-dependent HDACs, SIRT1, a member of the class III HDAC family, is an NAD^+^-dependent deacetylase that links cellular metabolism with epigenetic regulation. SIRT1 has been reported to influence CYP19A1 expression through histone deacetylation and by regulating the activity of transcription factors involved in estrogen biosynthesis. Although the precise molecular mechanisms remain under investigation, these observations provide direct evidence that specific HDAC isoforms participate in the epigenetic regulation of aromatase expression [132,197].

The epigenetic regulation of aromatase has important biological and therapeutic implications. Aberrant HDAC activity may simultaneously alter CYP19A1 transcription, estrogen receptor signaling, and the expression of estrogen-responsive genes, thereby creating a molecular environment that promotes tumor progression and endocrine resistance. These mechanisms are particularly relevant in HR+ breast cancer, where intratumoral estrogen production remains a major driver of disease progression despite systemic estrogen deprivation [198].

The recognition that aromatase expression is under epigenetic control provides a strong rationale for combining aromatase inhibitors with HDACi. While AIs suppress estrogen biosynthesis, HDACi may restore a more permissive chromatin configuration, reactivate silenced tumor suppressor genes, and improve ER signaling. Consequently, simultaneous targeting of endocrine and epigenetic pathways has emerged as a promising strategy for overcoming resistance to endocrine therapy and enhancing therapeutic efficacy in HR+ breast cancer [199].

### 4.2. Convergent Regulation of Estrogen-Dependent Gene Expression

Estrogen-dependent gene expression is regulated through a complex interplay between endocrine signaling and epigenetic mechanisms that together determine the transcriptional activity of estrogen-responsive genes. While aromatase controls the local biosynthesis of estrogens within the breast tumor microenvironment, HDACs regulate chromatin accessibility and the activity of transcriptional complexes associated with the ER. Rather than functioning as independent pathways, these mechanisms converge to modulate cell proliferation, differentiation, apoptosis, and therapeutic response in HR+ breast cancer [200,201].

ER+ breast cancer is characterized by substantial molecular and genetic heterogeneity, with recurrent alterations affecting ER signaling, growth-factor pathways, and cell-cycle regulation. Among these alterations, activation of the PI3K/Akt/mTOR pathway is particularly relevant, as it can result from mutations or other genomic changes affecting components of this signaling network and can promote tumor-cell proliferation, survival, and adaptation to endocrine therapy [94,96,97]. ESR1 mutations represent another important alteration, particularly in advanced and previously treated ER+ disease, where they may promote ligand-independent ER activity and sustain ER-dependent transcription despite reduced estrogen availability [95,98,99]. Together, these alterations contribute to the ability of ER+ tumors to maintain proliferative signaling under endocrine pressure [196,198].

PIK3CA-driven PI3K signaling is especially relevant because growth-factor and ER-dependent pathways converge functionally on cell-cycle control. Activation of PI3K/Akt signaling can reinforce proliferative and survival programs and interact with ER-mediated transcription, while ER and growth-factor signaling converge on CDK4/6 activity. Increased CDK4/6 activity promotes Rb phosphorylation, releases E2F transcription factors from Rb-mediated repression, and activates genes required for G1/S cell-cycle progression [151,152,153]. Thus, oncogenic signaling through the PI3K pathway may support tumor progression not only by enhancing cell survival but also by facilitating Rb/E2F-dependent cell-cycle escape. This crosstalk provides an important mechanistic link between genetic alterations, ER signaling, and proliferative control in ER+ breast cancer.

Importantly, these molecular alterations are not uniformly distributed across all ER+ tumors, and their biological and therapeutic relevance depends on tumor subtype, disease stage, and previous treatment exposure [196,198].

The biological effects of estrogens are primarily mediated by ERα, a ligand-activated transcription factor encoded by the ESR1 gene. Upon estrogen binding, ERα undergoes conformational changes that promote receptor dimerization, nuclear translocation, and binding to estrogen response elements located within the promoter regions of target genes. This interaction initiates the recruitment of numerous transcriptional co-regulators, including co-activators, co-repressors, chromatin-remodeling complexes, and histone-modifying enzymes, ultimately determining whether gene transcription is activated or suppressed. Consequently, the transcriptional output of ER signaling depends not only on estrogen availability but also on the epigenetic landscape surrounding estrogen-responsive genes [202,203].

Histone acetylation represents one of the most important epigenetic mechanisms controlling ER-mediated transcription. HATs, such as p300/CBP (CREB-binding protein) and p300/PCAF (CBP-associated factor), catalyze the acetylation of lysine residues within histone tails, resulting in chromatin relaxation and enhanced accessibility of transcription factors to DNA. Conversely, HDACs remove acetyl groups from histones, promoting chromatin condensation and transcriptional repression. This dynamic balance between acetylation and deacetylation determines the expression of numerous genes involved in cell-cycle progression, apoptosis, DNA repair, and cellular differentiation [204,205].

In addition to regulating histone acetylation, HDACs directly modulate the activity of several non-histone proteins involved in estrogen signaling. ERα and its associated transcriptional machinery are subject to reversible post-translational modifications, including acetylation, which can influence receptor function and transcriptional activity. HDAC-mediated deacetylation of ERα-associated regulatory proteins can therefore alter receptor-dependent transcription and the expression of estrogen-responsive genes. Moreover, HDACs regulate the activity of several transcription factors, including NF-κB, STAT3, activator protein 1 (AP-1), specificity protein 1 (SP1), and p53, many of which interact with signaling networks relevant to breast cancer progression and therapeutic response [12,204].

Efficient ER-dependent transcription also requires the coordinated recruitment of transcriptional co-regulators. Co-activator complexes, including members of the steroid receptor coactivator family, possess intrinsic histone acetyltransferase activity or recruit additional HAT-containing complexes that facilitate chromatin opening and transcriptional activation. In contrast, transcriptional co-repressors such as NCoR and SMRT recruit HDAC-containing complexes, leading to histone deacetylation and suppression of ER target genes [203]. The dynamic exchange between co-activator and co-repressor complexes is essential for maintaining physiological estrogen signaling, whereas disruption of this balance contributes to endocrine resistance [206].

Increasing evidence indicates that aberrant HDAC expression profoundly alters the transcriptional program of ER+ breast cancer cells. Overexpression of class I HDACs, particularly HDAC1, HDAC2, and HDAC3, has been associated with reduced expression of tumor suppressor genes, enhanced proliferative signaling, and poor clinical outcome [161]. HDAC-mediated chromatin remodeling may also silence genes involved in endocrine responsiveness while simultaneously activating compensatory signaling pathways, including PI3K/Akt/mTOR, MAPK (mitogen-activated protein kinase)/ERK, and growth factor receptor-mediated signaling. These adaptive changes enable tumor cells to maintain proliferation despite estrogen deprivation induced by AIs [207].

The convergence of estrogen signaling and epigenetic regulation extends beyond chromatin remodeling. HDACs also influence the expression and activity of pioneer transcription factors such as FOXA1 and GATA3 (GATA-binding protein 3), which facilitate ERα binding to chromatin and define lineage-specific transcriptional programs in luminal breast cancer. Alterations in these regulatory networks may reprogram ER-dependent gene expression, promote cellular plasticity, and facilitate disease progression [208,209].

Importantly, accumulating evidence suggests that epigenetic modifications are reversible, providing a strong rationale for therapeutic intervention. HDACi restore histone acetylation, reactivate silenced tumor suppressor genes, and modify the transcriptional activity of ER-associated complexes, thereby enhancing sensitivity to endocrine therapy. Experimental studies have demonstrated that HDAC inhibition increases the expression of ER-regulated genes, promotes cell-cycle arrest, induces apoptosis, and suppresses tumor cell proliferation. These effects are particularly relevant in endocrine-resistant breast cancer, where epigenetic silencing rather than irreversible genetic alterations often underlies therapeutic failure [204,210].

Collectively, these observations indicate that estrogen-dependent gene expression is governed by an integrated regulatory network involving estrogen biosynthesis, ER signaling, chromatin remodeling, and transcriptional regulation. The convergence of these pathways provides the biological basis for combining AIs with HDACi, as simultaneous targeting of endocrine and epigenetic mechanisms may enhance endocrine responsiveness, delay or overcome resistance, and improve therapeutic efficacy in HR+ breast cancer [211].

### 4.3. The Role of HDACs in Treatment Resistance to Aromatase Inhibitors

Overcoming therapeutic resistance remains a major challenge in improving outcomes for patients with cancer. Two promising approaches include the development of combination therapies and the identification of novel treatments that directly target the molecular pathways involved in resistance. By combining agents with distinct mechanisms of action, combination therapies can enhance treatment efficacy while simultaneously targeting multiple survival pathways exploited by cancer cells to evade therapeutic pressure [212]. This strategy may be particularly valuable when compensatory signaling pathways enable tumor cells to persist despite treatment. Furthermore, the selection of rational combination therapies can be guided by precision medicine approaches, taking into account patient-specific genetic alterations, molecular profiles, and dysregulated signaling pathways to identify the most appropriate therapeutic strategy [213].

Although aromatase AIs represent a highly effective endocrine therapeutic strategy for hormone HR+ breast cancer, the development of intrinsic or acquired resistance remains one of the major limitations of long-term treatment efficacy [74,214]. Resistance to estrogen deprivation is a complex and multifactorial process involving genetic alterations, activation of alternative signaling pathways, changes in ER activity, and extensive epigenetic reprogramming. Increasing evidence indicates that HDACs contribute significantly to this adaptive response by modifying chromatin structure and regulating transcriptional programs that allow tumor cells to survive under conditions of reduced estrogen availability [204,215].

Unlike genetic mutations, epigenetic alterations are reversible and provide tumor cells with the ability to rapidly adapt to therapeutic pressure. HDAC-mediated chromatin remodeling represents an important mechanism of transcriptional plasticity in endocrine-resistant breast cancer [216]. By promoting histone deacetylation and chromatin condensation, HDACs can suppress the expression of genes involved in cell-cycle control, apoptosis, and endocrine responsiveness, while simultaneously facilitating the activation of alternative survival pathways. Consequently, increased HDAC activity may enable breast cancer cells to maintain proliferative capacity despite inhibition of estrogen biosynthesis by AIs [204].

One of the most important mechanisms involved in endocrine resistance is the alteration of ER signaling. In the presence of aromatase inhibition, breast cancer cells may undergo adaptive changes that reduce their dependence on estrogen while preserving ER-mediated transcriptional activity [217]. HDACs regulate ERα function through multiple mechanisms, including modulation of receptor acetylation status, interaction with transcriptional co-regulators, and remodeling of ER-associated chromatin regions. Increased recruitment of HDAC-containing corepressor complexes may alter ER-dependent gene expression, whereas selective inhibition of HDAC activity can restore transcriptional programs associated with endocrine sensitivity [203].

HDACs also contribute to resistance by regulating the expression and activity of ER-associated transcription factors and chromatin regulators. Proteins such as FOXA1 and GATA3, which are essential for ERα binding to chromatin and maintenance of luminal breast cancer identity, are influenced by epigenetic mechanisms. Alterations in these regulatory networks may modify the genomic landscape of ER signaling, resulting in transcriptional reprogramming and the emergence of tumor cell populations capable of proliferating independently of estrogen stimulation [209,218].

In addition to alterations in ER signaling, endocrine-resistant tumors frequently activate alternative growth and survival pathways that compensate for estrogen deprivation. The PI3K/Akt/mTOR and MAPK/ERK signaling cascades are among the most important pathways involved in this adaptive response. HDACs regulate the transcription of multiple genes associated with these pathways and may facilitate their activation through epigenetic remodeling. For example, increased HDAC activity has been associated with enhanced survival signaling, resistance to apoptosis, and increased cellular proliferation, thereby reducing the effectiveness of endocrine therapies [219].

Cross-talk between estrogen signaling and growth factor receptor pathways, including HER2 signaling, represents another important mechanism of resistance. Activation of HER2 and related receptor tyrosine kinases can maintain tumor growth despite estrogen suppression by AIs [211]. HDAC-dependent regulation of receptor expression, downstream signaling molecules, and transcriptional responses may contribute to this bypass mechanism. Therefore, HDAC-mediated epigenetic regulation represents a common molecular mechanism linking endocrine resistance with activation of alternative oncogenic pathways [204].

Another important aspect of HDAC involvement in treatment resistance is their role in EMT and cancer cell plasticity. EMT is associated with reduced epithelial characteristics, increased migratory capacity, and acquisition of stem cell-like properties, all of which contribute to tumor progression and therapeutic resistance. HDACs regulate EMT-associated transcription factors, including Snail (snail family transcriptional repressor 1), Slug (snail family transcriptional repressor 2), and ZEB1 (zinc finger E-box-binding homeobox 1), through chromatin remodeling and transcriptional control. Increased HDAC activity may therefore promote the emergence of resistant cellular subpopulations with enhanced survival capacity and reduced sensitivity to endocrine therapy [220,221].

Cancer stem cell (CSC)-like populations represent another reservoir of endocrine-resistant cells. These cells exhibit self-renewal capacity, enhanced DNA repair mechanisms, and increased resistance to conventional therapies. HDACs contribute to CSC maintenance by regulating genes involved in stemness, differentiation, and survival. Inhibition of HDAC activity has been shown to reduce stem-like characteristics and increase the responsiveness of tumor cells to anticancer therapies, supporting the potential role of HDACi in overcoming resistance mechanisms [222].

The involvement of HDACs in multiple resistance pathways provides a strong biological rationale for their therapeutic targeting in combination with AIs. By restoring chromatin accessibility, reactivating silenced genes involved in endocrine response, and suppressing compensatory survival mechanisms, HDACi may enhance the efficacy of estrogen deprivation strategies [161]. This concept has been supported by preclinical studies demonstrating increased sensitivity to endocrine therapy following HDAC inhibition, as well as by clinical investigations evaluating combinations of HDACi with endocrine agents [223].

Overall, HDACs act as important regulators of endocrine resistance by integrating epigenetic alterations with ER signaling and alternative oncogenic pathways. Their ability to modulate multiple mechanisms involved in treatment failure highlights HDACs as promising therapeutic targets for improving responses to aromatase inhibition. Understanding the specific HDAC isoforms involved in resistance and identifying predictive biomarkers will be essential for the successful implementation of combined epigenetic and endocrine therapeutic approaches in breast cancer [11,224].

### 4.4. Therapeutic Synergy

Combination therapies offer several potential advantages, including synergistic or complementary effects that may produce greater therapeutic efficacy than monotherapy. The use of agents with different mechanisms of action may also allow lower doses of individual drugs while maintaining antitumor activity, potentially reducing treatment-related toxicity and adverse effects [225]. In parallel, emerging therapeutic strategies targeting resistance-associated pathways aim to address the adaptive nature of cancer cells and counteract the molecular mechanisms that enable tumors to develop resistance to standard treatments [226].

The increasing understanding of the molecular interactions between estrogen signaling and epigenetic regulation has provided a strong rationale for combining AIs with HDACi in HR+ breast cancer. While AIs effectively suppress estrogen biosynthesis by inhibiting aromatase activity and reducing estrogen availability, they do not directly target the epigenetic mechanisms responsible for transcriptional adaptation, tumor cell plasticity, and altered hormone responsiveness. Conversely, HDACi modify chromatin structure and regulate the expression of multiple genes involved in cell proliferation, apoptosis, differentiation, and endocrine sensitivity. Therefore, simultaneous inhibition of estrogen production and epigenetic regulation represents a complementary therapeutic strategy capable of targeting distinct but interconnected pathways involved in breast cancer progression.

The biological rationale for this combination is based on the concept that estrogen deprivation alone may be insufficient to completely suppress tumor growth because breast cancer cells can undergo transcriptional and epigenetic adaptations. HDAC inhibition may counteract these adaptations by restoring chromatin acetylation, increasing the accessibility of estrogen-responsive genes, and reestablishing a transcriptional profile associated with endocrine sensitivity. In this context, HDACi may function as sensitizing agents that enhance the antitumor activity of aromatase inhibition rather than acting solely as independent cytotoxic compounds [11].

The combination of an AI and an HDAC inhibitor may result in more potent inhibition of tumor growth, induction of apoptosis, and suppression of resistance mechanisms. Although there is no direct enzymatic interaction between the two, they are functionally linked through epigenetic regulation, control of estrogen-dependent gene expression, and shared mechanisms of endocrine resistance. Emerging preclinical and clinical evidence supports a functional interplay between HDAC inhibition and aromatase-targeted therapy, demonstrating synergistic antitumor effects and the potential to overcome endocrine resistance [191].

Preclinical and clinical studies have investigated several HDACi, particularly entinostat, panobinostat, vorinostat, and tucidinostat, in combination with endocrine therapy. These agents may enhance endocrine responsiveness through restoration of hormone-related transcriptional programs, suppression of adaptive survival pathways, induction of cell-cycle arrest and apoptosis, and modulation of mechanisms associated with acquired AI resistance. The available evidence supporting this therapeutic strategy is summarized below according to its translational level, from in vitro studies to preclinical in vivo models and clinical trials [227,228,229,230,231].

Initial evidence supporting combined aromatase and HDAC inhibition was obtained in breast cancer cell models. These studies demonstrated that HDAC inhibition can modify estrogen receptor and aromatase expression, suppress signaling pathways involved in acquired endocrine resistance, and restore sensitivity to aromatase inhibition. Particularly relevant findings were obtained in aromatase-expressing MCF-7-derived models rendered resistant to long-term AI exposure, in which HDAC inhibition reduced proliferative capacity and reversed molecular features associated with resistance [227,232]. The main in vitro studies are summarized in Table 3.

Taken together, these in vitro findings indicate that HDAC inhibition may enhance aromatase-targeted therapy through at least two complementary mechanisms. First, epigenetic reactivation of ERα and aromatase can restore hormone dependence and increase responsiveness to aromatase inhibition. Second, in cells with acquired AI resistance, HDAC inhibitors can suppress adaptive survival pathways, including HER2- and NF-κB-associated signaling, while promoting cell-cycle arrest and apoptosis [228]. These cellular observations provided the mechanistic foundation for subsequent in vivo studies and clinical evaluation of HDAC inhibitor–AI combinations.

Preclinical evidence provides direct support for combining aromatase inhibition with HDAC blockade, particularly in models of acquired endocrine resistance. In aromatase-expressing ER+ breast cancer xenografts that had developed resistance to letrozole or exemestane, HDAC inhibition restored or enhanced the antitumor activity of aromatase inhibitors. These effects were associated with suppression of compensatory HER2 and NF-κB signaling, restoration of endocrine responsiveness, induction of cell-cycle arrest and apoptosis, and reduction in tumor-initiating cell populations [227,228]. The principal in vivo studies evaluating these therapeutic interactions are summarized in Table 4.

Collectively, these preclinical findings indicate that HDAC inhibition can enhance or restore the antitumor efficacy of AIs in endocrine-resistant breast cancer. Rather than acting solely through nonspecific cytotoxicity, HDACi appear to reverse adaptive mechanisms associated with AI resistance, including HER2- and NF-κB-mediated signaling, altered ER-dependent transcription, and persistence of tumor-initiating cell populations. These observations provided a mechanistic basis for the subsequent clinical evaluation of HDACi in combination with AIs in HR+ breast cancer [227,228].

The encouraging preclinical findings prompted the clinical evaluation of HDACi in combination with AIs, particularly in patients with HR+ advanced breast cancer who had developed resistance to previous endocrine therapy. Most clinical experience has been obtained with entinostat or tucidinostat combined with exemestane, whereas panobinostat has been investigated in an earlier-phase trial with letrozole. The principal clinical studies evaluating combined HDAC and aromatase inhibition are summarized in Table 5.

Clinical evidence for combined HDAC and aromatase inhibition remains heterogeneous. Differences in efficacy across trials may reflect variations in HDAC inhibitor properties, patient populations, previous endocrine exposure, and molecular determinants of endocrine resistance. These findings support the need for predictive biomarkers and improved patient selection for endocrine–epigenetic combination strategies [229,231]. Collectively, these findings suggest that the therapeutic efficacy of HDAC–AI combinations may depend on the characteristics of the HDAC inhibitor, patient population, prior endocrine exposure, and molecular determinants of endocrine resistance. Identification of predictive biomarkers therefore remains essential for selecting patients most likely to benefit from this therapeutic strategy.

Particular attention was given to studies evaluating combined aromatase and HDAC inhibition in ER+/HER2-negative breast cancer and in models of acquired AI resistance. HER2 signaling was considered when reported as an adaptive mechanism of endocrine resistance rather than as evidence derived from primary HER2-positive disease. The available data support a functional therapeutic interplay between estrogen deprivation and HDAC-targeted epigenetic modulation, while the heterogeneous clinical responses emphasize the need for biomarker-guided patient selection.

Thus, the interplay between estrogen signaling and epigenetic regulation provides a compelling rationale for combining aromatase inhibitors with HDACi; however, the heterogeneous clinical responses observed to date underscore the need for biomarker-driven patient selection and further evaluation of optimal therapeutic combinations.

## 5. Conclusions and Future Perspectives

AIs have substantially improved the treatment of ER+ breast cancer; however, their clinical effectiveness remains limited in some patients, particularly because of the development of therapeutic resistance. Consequently, ongoing research is focused on developing strategies that achieve more effective suppression of estrogen signaling and on combining AIs with complementary therapeutic approaches to improve clinical outcomes. These approaches include the investigation of novel strategies targeting aromatase activity alongside alternative hormone-dependent or hormone-independent signaling pathways, as well as the integration of endocrine therapy with molecularly targeted agents. Advances in the characterization of breast cancer molecular profiles have further supported the development of personalized treatment strategies. In this context, current research increasingly focuses on identifying biomarkers associated with treatment response, resistance, or limited therapeutic benefit to facilitate patient selection and optimize treatment outcomes [237,238,239].

The integration of biomarkers into clinical practice is becoming increasingly important for predicting patient responses to AIs. Ongoing research aims to identify genetic and molecular markers capable of distinguishing patients who are most likely to derive benefit from AI-based therapy from those at higher risk of treatment failure. Such biomarker-guided approaches may improve therapeutic efficacy while avoiding unnecessary exposure to ineffective treatments. Moreover, the application of precision medicine enables treatment strategies to be tailored to individual genetic profiles and tumor characteristics, with the potential to improve clinical outcomes and minimize treatment-related toxicity [240,241].

Future research should prioritize a more comprehensive understanding of the mechanisms underlying resistance to AIs, including the activation of alternative signaling pathways, adaptive alterations in ER signaling, epigenetic reprogramming, and changes within the tumor microenvironment. Increasing evidence indicates that epigenetic dysregulation plays an important role in the development of endocrine resistance, providing a rationale for investigating HDACi as potential therapeutic partners for AIs. By modulating chromatin accessibility and potentially restoring the expression of estrogen-responsive and tumor-suppressor genes, HDAC inhibition may increase the sensitivity of breast cancer cells to estrogen deprivation resulting from aromatase blockade. Consequently, combining HDACi with AIs represents a promising therapeutic strategy for overcoming endocrine resistance in HR+ breast cancer. However, the clinical implementation of such combinations will require the identification and validation of predictive biomarkers to select patients most likely to benefit and to support the development of personalized treatment approaches [114].

An important challenge in developing AI–HDAC inhibitor combinations is achieving therapeutic efficacy while minimizing toxicity. HDACi can induce adverse effects such as fatigue, thrombocytopenia, gastrointestinal disturbances, and hematological toxicity, which may limit their long-term use. Therefore, current research focuses on developing selective HDACi, optimizing dosing schedules, and identifying biomarkers capable of predicting which patients are most likely to benefit from combination treatment [242].

Future therapeutic strategies may involve the integration of aromatase inhibition and HDAC targeting with other precision medicine approaches. The identification of molecular signatures associated with endocrine sensitivity, HDAC dependency, and epigenetic vulnerability may allow improved patient stratification and personalized treatment selection. Furthermore, combination strategies involving HDAC inhibitors, CDK4/6 inhibitors, PI3K/Akt/mTOR pathway inhibitors, or other targeted agents may provide additional opportunities to overcome treatment limitations in advanced HR+ breast cancer [243].

Clinical outcomes in oncology may be further improved through the integration of emerging therapeutic approaches, including gene-editing technologies, epigenetic therapies, and artificial intelligence-based strategies. A deeper understanding of how epigenetic alterations regulate hormone receptor expression and influence tumor behavior may reveal new therapeutic opportunities. In particular, combining AIs with epigenetic agents could represent a promising strategy for restoring endocrine sensitivity and overcoming resistance in tumors that have become refractory to conventional endocrine therapy [244]. Artificial intelligence-based analyses may improve patient stratification and treatment planning by identifying complex molecular patterns associated with therapeutic response and resistance. Machine learning algorithms can integrate tumor-specific molecular and clinical characteristics to support the selection of personalized treatment strategies and potentially identify optimal therapeutic combinations. These approaches may therefore offer new opportunities for precision medicine and more individualized management of breast cancer [245].

Achieving optimal treatment adherence and improving therapeutic outcomes requires careful attention to patient concerns, effective communication, and ongoing clinical support. During long-term endocrine therapy, regular monitoring of treatment-related adverse effects and the implementation of proactive management strategies are essential to maintain adherence, minimize treatment interruptions, and maximize therapeutic benefit [238,246]. Ongoing research aims to develop more selective AIs with improved therapeutic efficacy and more favorable safety profiles. Advances in drug-delivery technologies, including long-acting injectable formulations and optimized oral preparations, may improve drug bioavailability, treatment adherence, and patient convenience. In addition, integrating nutritional strategies with psychological and rehabilitative interventions may provide comprehensive supportive care, potentially improving treatment tolerance, overall outcomes, and quality of life in patients receiving endocrine therapy [247,248].

The future of AI therapy in breast cancer treatment is highly promising, driven by the development of improved inhibitors, rational combination strategies, and approaches aimed at overcoming endocrine resistance. The integration of aromatase inhibition with epigenetic therapies, particularly HDAC-targeted approaches, represents a promising direction for improving therapeutic responses in HR+ breast cancer. Overall, the combination of AIs and HDACi represents a rational therapeutic approach based on the complementary regulation of estrogen biosynthesis and epigenetic control of gene expression. By simultaneously targeting hormonal stimulation and tumor-associated epigenetic alterations, this strategy has the potential to enhance endocrine therapy efficacy, delay resistance development, and improve outcomes in patients with HR+ breast cancer. Continued advances in molecular oncology and precision medicine may ultimately lead to more effective and individualized treatment strategies [249,250].

## Figures and Tables

**Figure 1:**
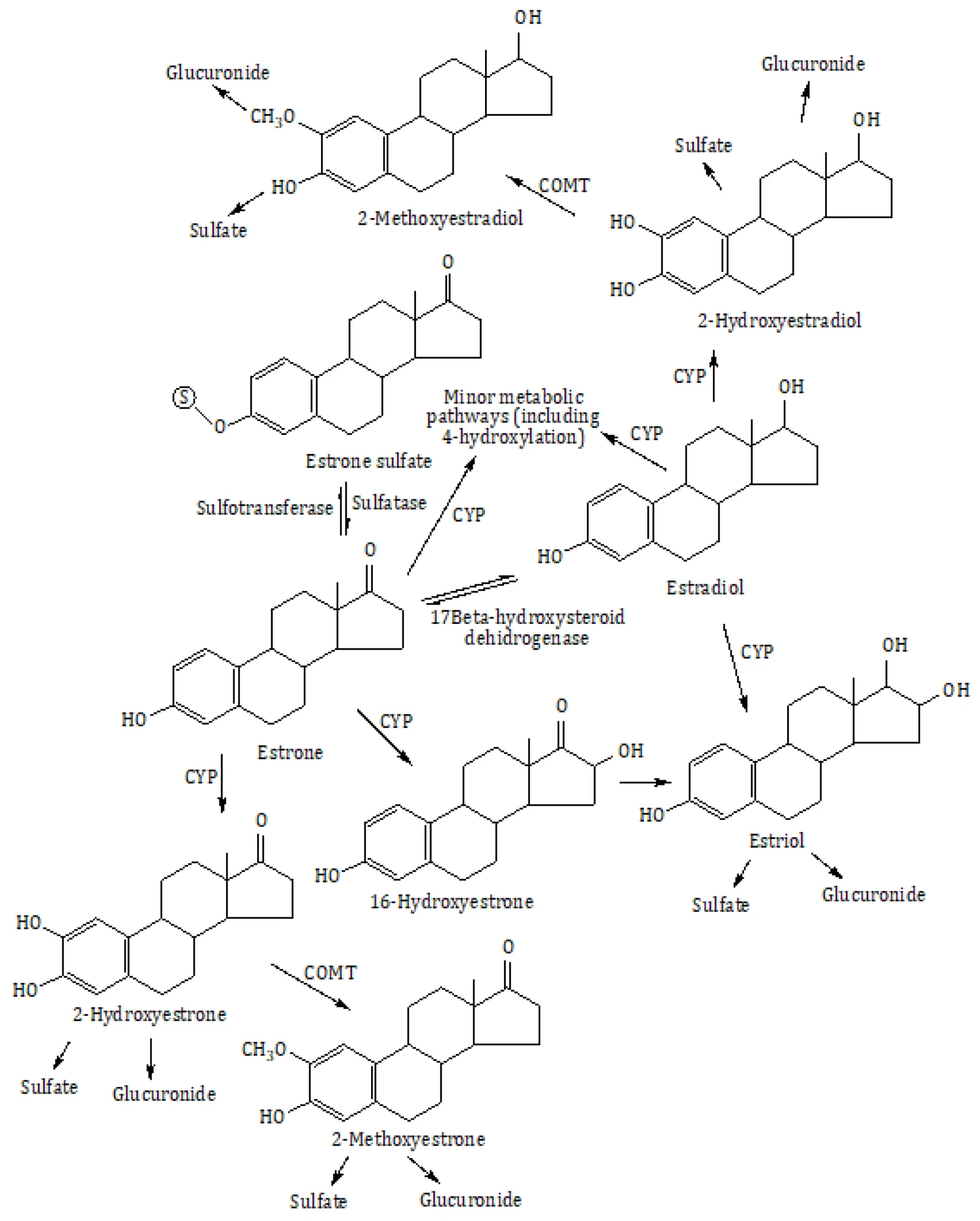
Interconversion reactions catalyzed by 17 beta-hydroxysteroid dehydrogenase. Conversion to catechol estrogens, conversion to methoxyestrogens, and conjugation with glucuronic acid or sulfate.

**Figure 2:**
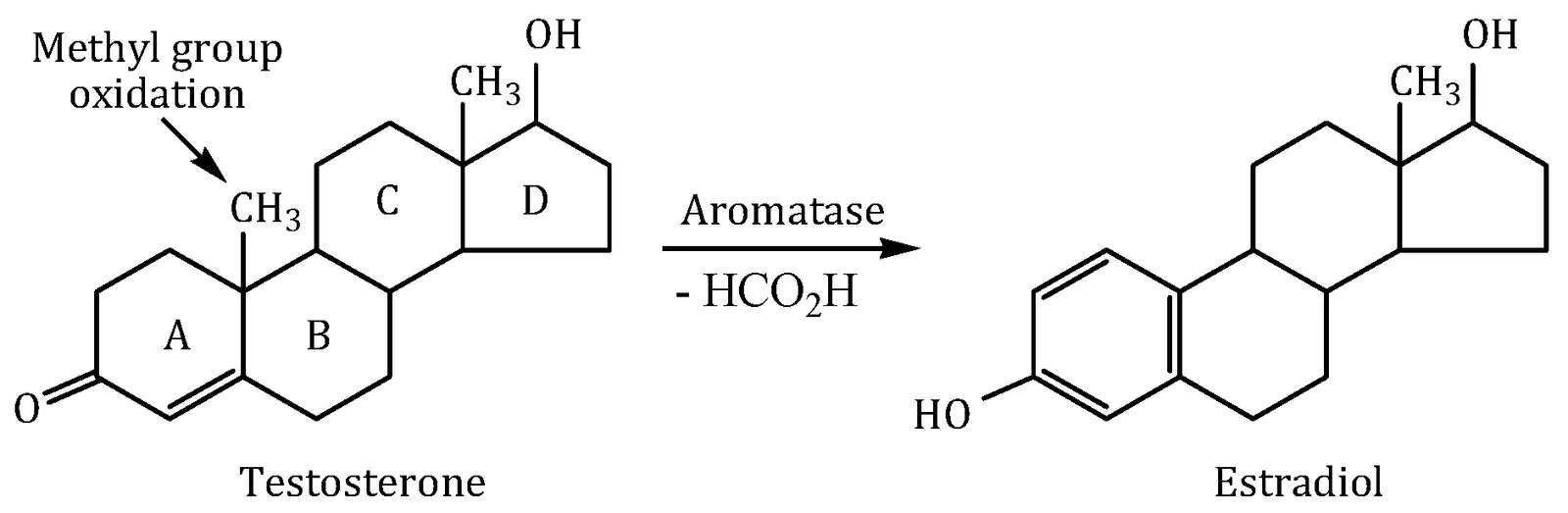
Schematic representation of the aromatization reaction (indicating the methylation position).

**Figure 3:**
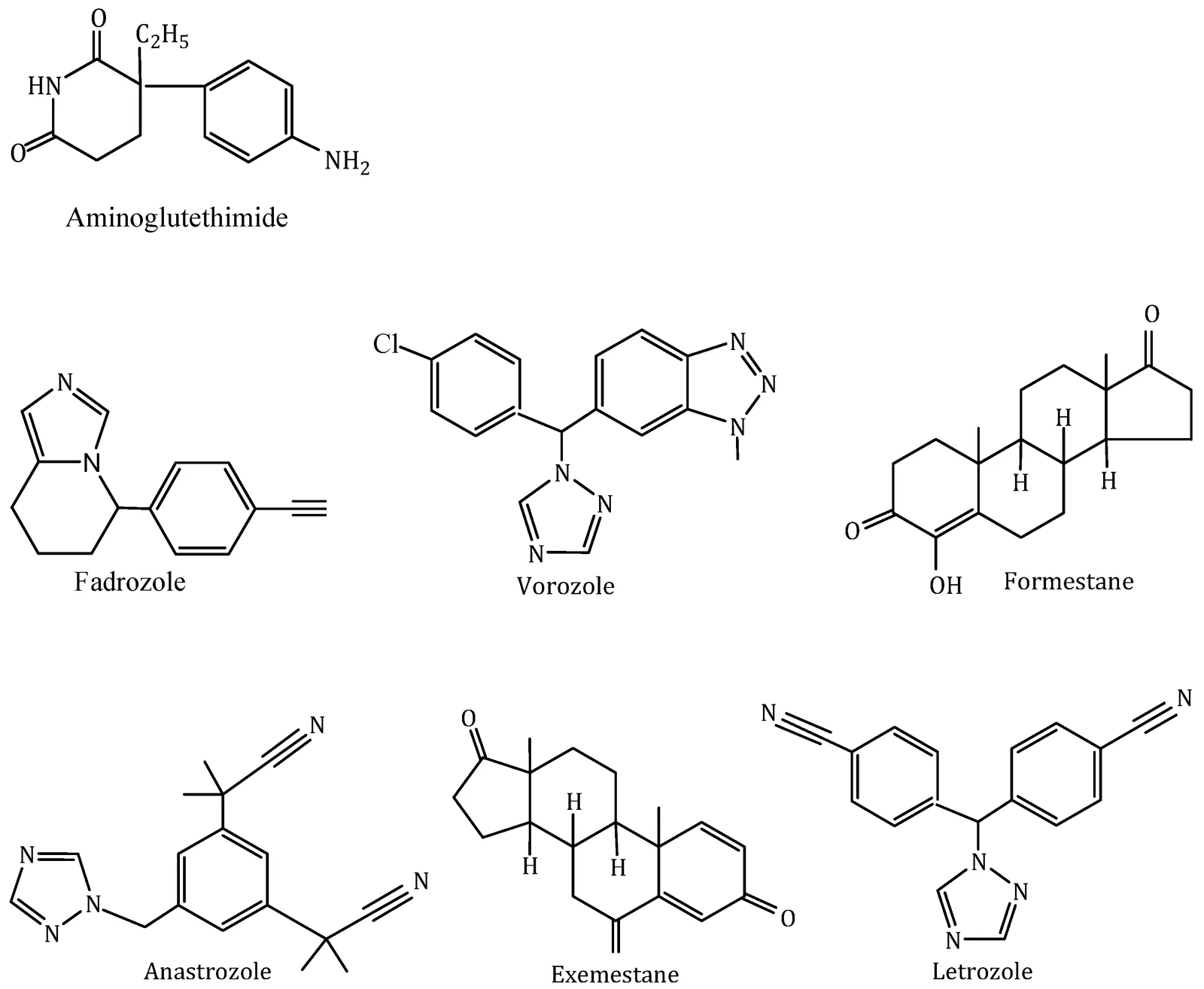
Chemical structures of representative aromatase inhibitors.

**Figure 4:**
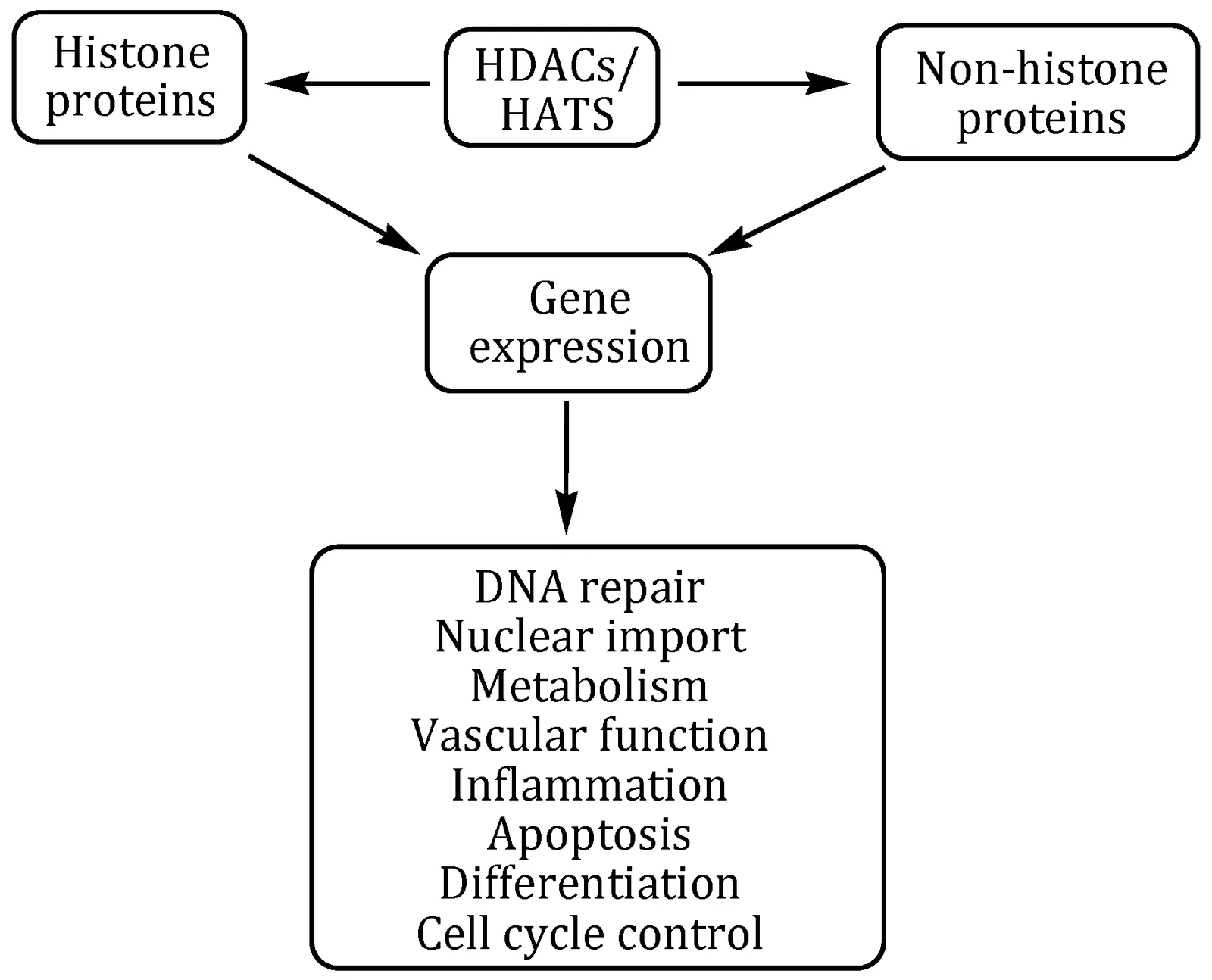
Regulation of gene expression and cellular functions by acetylation and deacetylation of histone and non-histone proteins.

**Figure 5:**
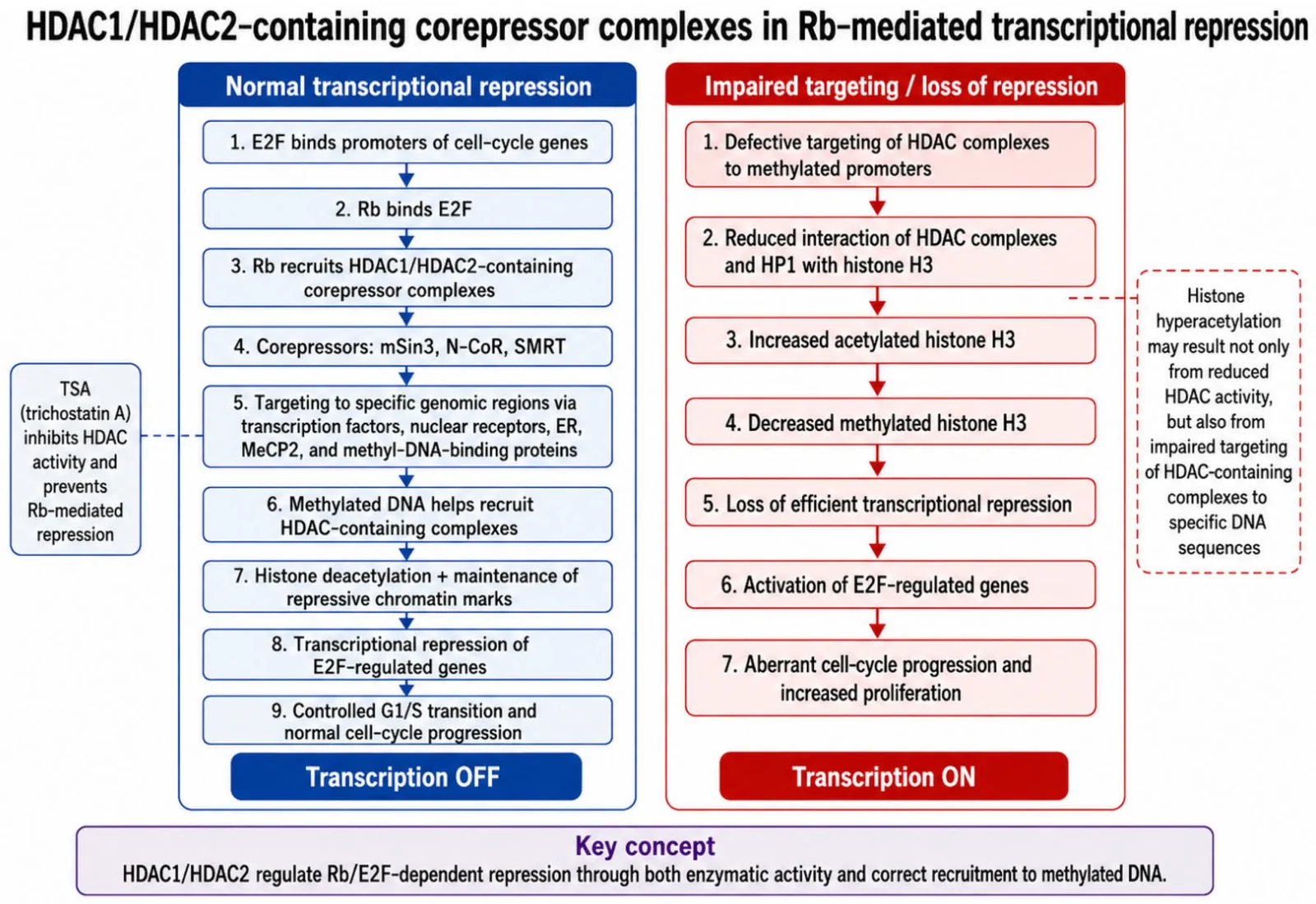
Rb/E2F-dependent transcriptional repression through HDAC1/HDAC2-containing corepressor complexes and its disruption in cancer. **Abbreviations:** HDAC1/2, histone deacetylases 1 and 2; Rb, retinoblastoma protein; E2F, E2 transcription factor; mSin3, mammalian Sin3 corepressor; N-CoR, nuclear receptor corepressor; SMRT, silencing mediator for retinoid and thyroid hormone receptors; MeCP2, methyl-CpG-binding protein 2; TSA, trichostatin A; G1/S, transition from G1 phase to S phase of the cell cycle.

**Figure 6:**
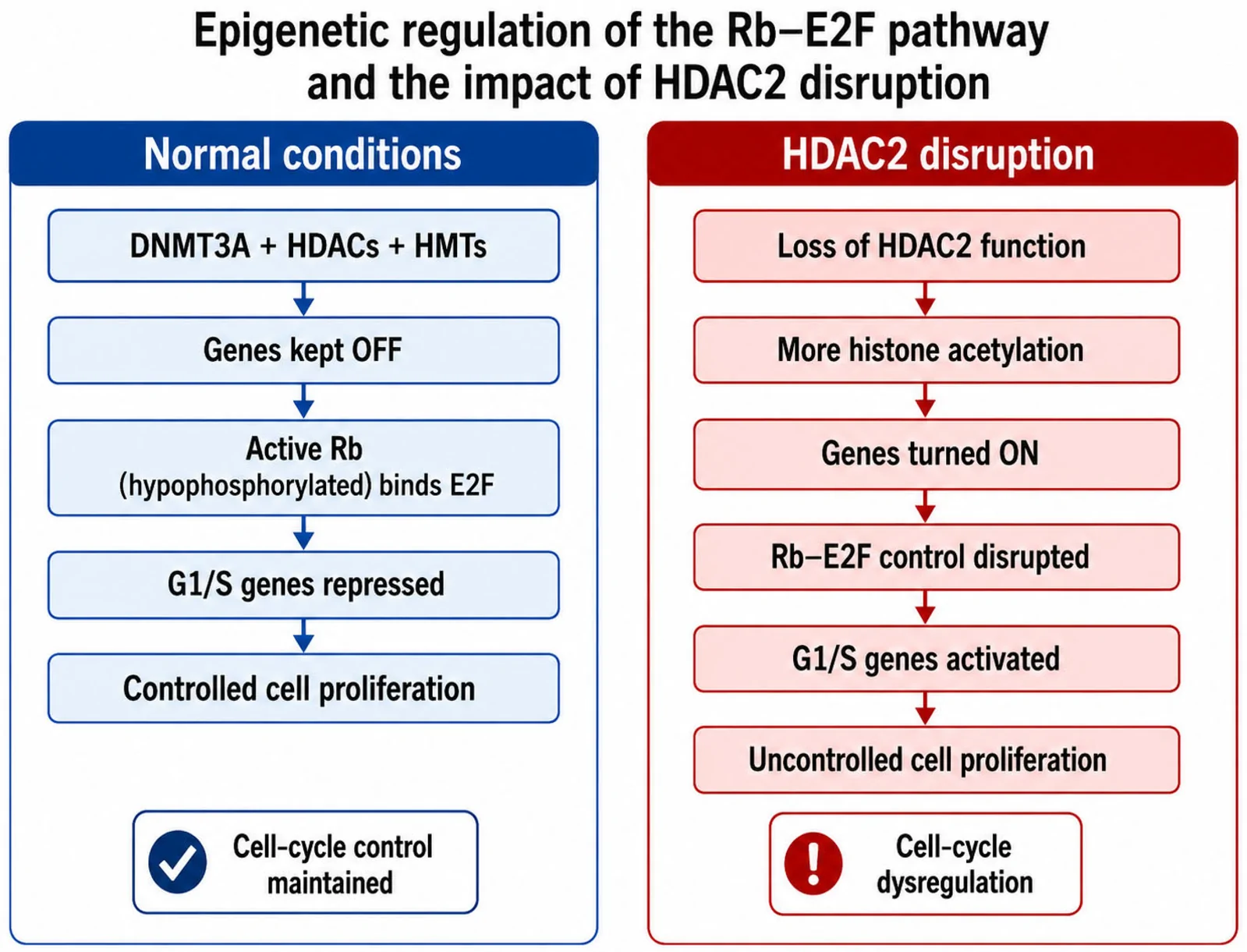
Epigenetic regulation of the Rb/E2F pathway under physiological conditions and following HDAC2 dysregulation. Under normal conditions, DNMT3A, HDACs, and HMTs contribute to the maintenance of transcriptional repression and appropriate cell-cycle control. Hypophosphorylated Rb binds E2F transcription factors, thereby repressing genes required for the G1/S transition and maintaining controlled cellular proliferation. In the setting of HDAC2 dysregulation, alterations in histone acetylation and gene expression may impair Rb/E2F-dependent transcriptional control, promote activation of G1/S-related genes, and facilitate aberrant cell-cycle progression. In breast cancer, these epigenetic alterations may cooperate with oncogenic signaling pathways and contribute to sustained proliferation, tumor progression, and therapeutic resistance. Abbreviations: DNMT3A, DNA methyltransferase 3A; HDACs, histone deacetylases; HDAC2, histone deacetylase 2; HMTs, histone methyltransferases; Rb, retinoblastoma protein; E2F, E2 transcription factor; G1/S, transition from G1 phase to S phase of the cell cycle.

**Figure 7:**
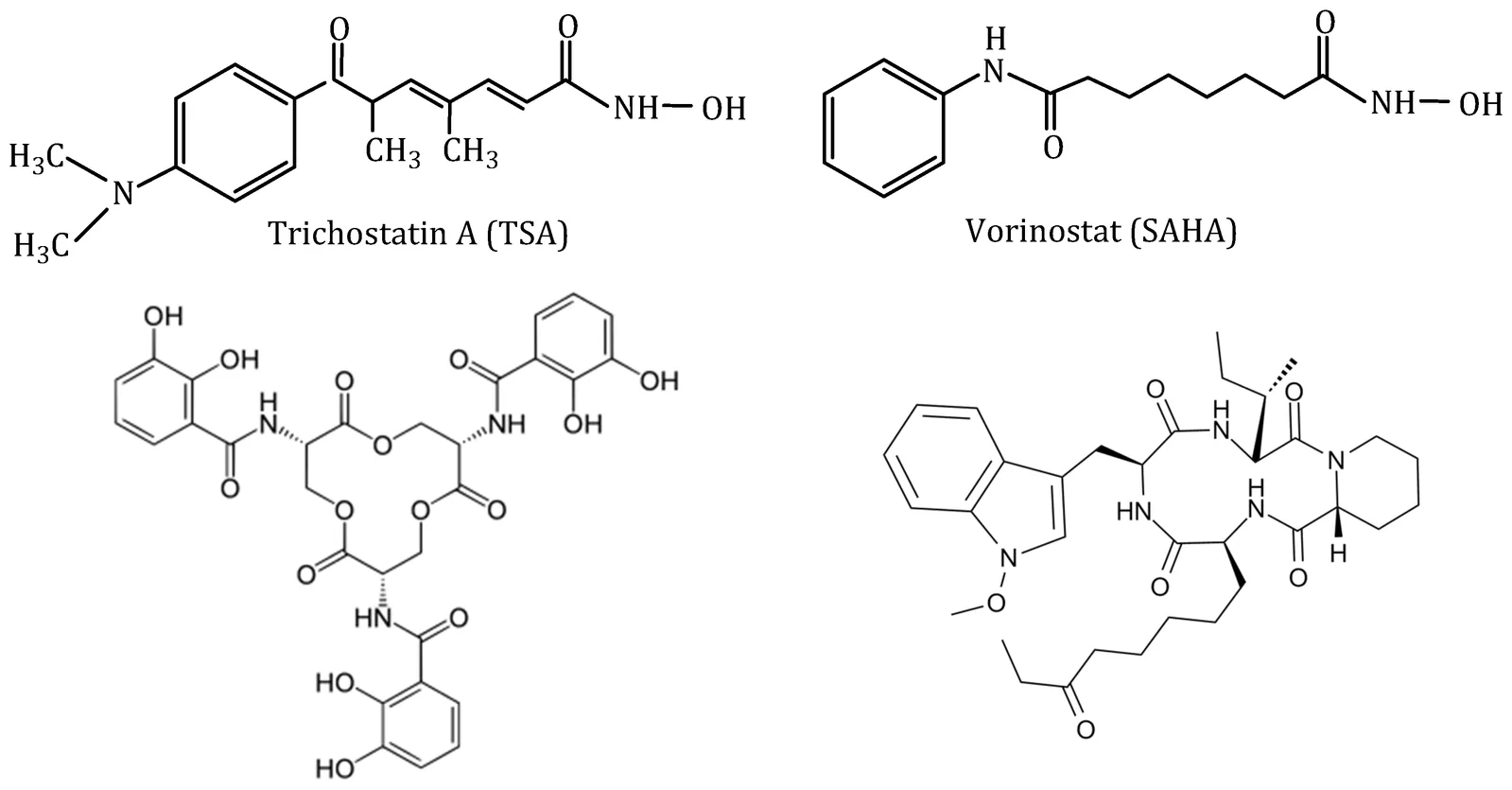
Structures of selected HDAC inhibitors.

**Table 1:** Tissue-specific distribution and physiological relevance of aromatase, highlighting the principal sites of estrogen production and the changes associated with menopausal status.

Tissue/Site	Aromatase-Related Estrogen Production	Main Estrogenic Contribution	Principal Target Tissues	Effect of Menopausal Status
Ovary	Major site of estrogen biosynthesis during reproductive life	Predominantly estradiol	Breast, uterus, bone and other estrogen-responsive tissues	Dominant source before menopause; ovarian estrogen production markedly decreases after menopause
Adipose tissue	Peripheral conversion of androgen precursors into estrogens	Predominantly estrone, with conversion to estradiol	Breast, bone and other peripheral estrogen-responsive tissues	Relative contribution becomes substantially greater after menopause
Breast tissue	Local aromatase permits intracrine/paracrine estrogen formation	Local estrone and estradiol production	Mammary epithelial and ER-positive breast cancer cells	Local estrogen synthesis becomes particularly relevant after menopause
Adrenal/peripheral tissues	Adrenal androgen precursors serve as substrates for peripheral aromatization	Estrone and estradiol	Multiple estrogen-responsive tissues	Increasing relative importance after menopause
Skin and blood vessels	Extra-gonadal aromatase supports local estrogen formation	Locally produced estrogens	Cutaneous and vascular tissues	Local production persists after menopause
Brain	Local aromatase supports tissue-specific estrogen synthesis	Mainly local estrogen production	Neuronal and glial cells	Primarily a local source throughout adult life
Placenta	Major aromatase-dependent estrogen production during pregnancy	High estrogen production during gestation	Maternal and fetal tissues	Related primarily to pregnancy rather than menopausal status

**Abbreviations:** ER, estrogen receptor.

**Table 2:** Classification of human histone deacetylases and representative inhibitors.

Class	Members	Representative Compounds
I	HDAC1, HDAC2, HDAC3, HDAC8	Vorinostat (SAHA), panobinostat (LBH589), belinostat (PXD 101), givinostat (ITF2357), abexinostat (PCI-24781), entinostat (MS-275), butyrate, 4-phenylbutyrate (4-PBA), valproic acid (VPA), trichostatin A (TSA), mocetinostat (MGCD0103), pracinostat (SB939), tucidinostat (chidamide, CS055/HBI-8000), romidepsin (FK228), m-carboxycinnamic acid bis-hydroxamide (CBHA) [157,165,166,167,168,169,170]
IIa	HDAC4, HDAC5, HDAC7, HDAC9	Vorinostat, panobinostat, belinostat, pracinostat, 4-phenylbutyrate, valproic acid, trichostatin A, dacinostat (LAQ824) [165,166,167]
IIb	HDAC6, HDAC10	Vorinostat, panobinostat, belinostat, pracinostat, givinostat, abexinostat, trichostatin A, dacinostat, tucidinostat [165,166,167,169]
III	SIRT1-SIRT7	Nicotinamide (NAM), tenovin-1, tenovin-6, sirtinol, selisistat (EX-527) [171,172]
IV	HDAC11	Vorinostat, panobinostat, belinostat, pracinostat, trichostatin A, dacinostat [165,166,167]

**Table 3:** In vitro evidence supporting HDAC inhibition in ER-positive and aromatase inhibitor-resistant breast cancer models.

Breast Cancer Cell Model	Hormone/AI Sensitivity	Aromatase Inhibitor	HDAC Inhibitor	Main In Vitro Findings	Mechanistic Significance	Study
MDA-MB-231 human breast cancer cells; additional experiments with MCF-7Ca	Initially ERα-negative; epigenetically endocrine-insensitive	Letrozole	Entinostat (MS-275); Vorinostat (SAHA) also evaluated	Entinostat induced ERα and aromatase expression and aromatase activity. MDA-MB-231 cells were poorly responsive to letrozole alone (IC50 > 10 μM), whereas addition of entinostat markedly sensitized cells to letrozole (IC50 ≈ 6.17 nM).	HDAC inhibition reverses epigenetic silencing of ERα/aromatase, creating an estrogen-responsive phenotype and restoring susceptibility to aromatase inhibition.	[233]
LTLT-Ca, derived from aromatase-expressing MCF-7Ca cells after long-term letrozole exposure	Acquired letrozole-resistant, ER-related model	Letrozole	Entinostat	Entinostat inhibited proliferation of letrozole-resistant cells and restored responsiveness to letrozole. Combination treatment was associated with greater inhibition than continued AI exposure alone.	Resistance was associated with increased HER2 signaling; entinostat decreased HER2 expression/activity and altered downstream signaling, supporting epigenetic reversal of acquired AI resistance.	[227]
Aromatase-expressing breast cancer cell lines with acquired resistance to exemestane, letrozole or anastrozole	AI-resistant	Previous/existing exposure to exemestane, letrozole or anastrozole	Panobinostat (LBH589)	Panobinostat strongly inhibited proliferation of acquired AI-resistant cells in vitro, induced G2/M cell-cycle arrest and increased apoptosis.	HDAC inhibition suppresses survival mechanisms maintained after prolonged AI exposure; effects were associated particularly with suppression of NF-κB1 signaling.	[228]
Letrozole-resistant MCF-7Ca-derived cells	acquired letrozole resistance	Letrozole	Entinostat	Entinostat-containing treatment reduced growth and characteristics of the tumor-initiating cell population, including mammosphere-forming capacity. The greatest effects were observed in combination regimens incorporating entinostat and letrozole.	HDAC inhibition reduced HER2 expression and targeted a resistant/stem-like cell population potentially responsible for persistence during AI therapy.	[232]
MCF-7 human breast cancer cells	ER-positive	Letrozole	Vorinostat (SAHA)	The study examined cytotoxic responses to SAHA in MCF-7 cells and also evaluated combinations involving letrozole. HDAC inhibition promoted programmed cell-death pathways and modified the response to endocrine treatment.	Supports an interaction between HDAC inhibition, estrogen-dependent growth and cell-death signaling, although this study is less directly focused on AI-resistance reversal than the Sabnis studies.	[234]

Abbreviations: AI, aromatase inhibitor; ER, estrogen receptor; HDAC, histone deacetylase; HER2, human epidermal growth factor receptor 2; IC_50_, half-maximal inhibitory concentration.

**Table 4:** Preclinical in vivo evidence supporting HDAC inhibition in combination with aromatase-targeted therapy in endocrine-resistant breast cancer.

Experimental Model	Aromatase Inhibitor	HDAC Inhibitor	Treatment/ Comparison	Main Findings	Mechanistic Significance	Study
Ovariectomized athymic nude mice bearing aromatase-expressing MCF-7Ca xenografts with acquired letrozole resistance	Letrozole	Entinostat	Entinostat alone or entinostat + letrozole versus continued letrozole/control treatment	Entinostat restored responsiveness of letrozole-resistant tumors; entinostat + letrozole produced sustained tumor-growth suppression and was more effective than continued letrozole treatment.	HDAC inhibition reduced adaptive HER2 expression/ activity, modulated MAPK signaling, and restored ER-associated endocrine responsiveness.	[227]
Mouse xenografts derived from exemestane-resistant, aromatase-expressing breast cancer cells	Exemestane	Panobinostat (LBH589)	Exemestane-resistant tumors maintained on exemestane with or without panobinostat	Addition of panobinostat significantly inhibited tumor growth and reduced tumor weight; decreased Ki-67 and increased apoptosis/cleaved PARP were observed.	Panobinostat suppressed NF-κB1 signaling and promoted cell-cycle arrest and apoptosis, supporting reversal of an important acquired AI-resistance mechanism.	[228]

**Abbreviations:** AI, aromatase inhibitor; ER, estrogen receptor; HDAC, histone deacetylase; HER2, human epidermal growth factor receptor 2; Ki-67, proliferation marker protein; MAPK, mitogen-activated protein kinase; NF-κB1, nuclear factor κB subunit 1; PARP, poly(ADP-ribose) polymerase.

**Table 5:** Clinical evidence for aromatase inhibitor–HDAC inhibitor combinations in hormone receptor-positive advanced breast cancer.

Study/Phase	Population	N	Treatment	Key Efficacy Outcomes	Interpretation
ENCORE 301, Phase II [229]	Postmenopausal, ER+ locally recurrent/metastatic BC after nonsteroidal AI	130	Exemestane + entinostat vs. exemestane + placebo	PFS: 4.3 vs. 2.3 mo; OS: 28.1 vs. 19.8 mo; ORR: 6.3% vs. 4.6%	Improved PFS; exploratory OS benefit
Phase I [230]	Postmenopausal metastatic BC	12	Letrozole + panobinostat	2 partial responses; 5 stable disease	Feasible combination with preliminary activity
ACE, Phase III [231]	Postmenopausal HR+/HER2− advanced BC after endocrine therapy	365	Exemestane + tucidinostat vs. exemestane + placebo	PFS: 7.4 vs. 3.8 mo; ORR: 18% vs. 9%; CBR: 47% vs. 36%	Significant PFS and response improvement
E2112, Phase III [235]	HR+/HER2− advanced BC resistant to nonsteroidal AIs	608	Exemestane + entinostat vs. exemestane + placebo	PFS: 3.3 vs. 3.1 mo; OS: 23.4 vs. 21.7 mo; ORR: 5.8% vs. 5.6%	No significant PFS or OS benefit
Phase II, Japanese cohort [236]	HR+/HER2− advanced/recurrent BC	98	Exemestane + entinostat vs. exemestane + placebo	PFS: 5.8 vs. 3.3 mo	Numerical PFS improvement; not statistically significant
Phase III, Chinese cohort [155]	HR+ advanced BC after ≥1 endocrine therapy	354	Exemestane + entinostat vs. exemestane + placebo	PFS: 6.32 vs. 3.72 mo; ORR: 17.4% vs. 10.9%	Significant PFS improvement

**Abbreviations:** AI, aromatase inhibitor; BC, breast cancer; CBR, clinical benefit rate; ER+, estrogen receptor-positive; HDAC, histone deacetylase; HER2−, human epidermal growth factor receptor 2-negative; HR+, hormone receptor-positive; ORR, objective response rate; OS, overall survival; PFS, progression-free survival.

## Data Availability

No new data were created or analyzed in this study. Data sharing is not applicable to this article.

## Abbreviations

The following abbreviations are used in this manuscript:

ADAMTS8	ADAM metallopeptidase with thrombospondin type 1 motif 8
AF-2	activation function-2
AIs/AI	aromatase inhibitors/aromatase inhibitor
Akt	protein kinase B
AP-1	activator protein 1
AR	androgen receptor
ARHI	aplasia ras homolog I = DIRAS3
BCR-ABL	BCR::ABL1 fusion protein
Bmf	Bcl-2 modifying factor
BMI	body mass index
BRCA1 and BRCA2	breast cancer gene 1 and 2
CBP	CREB-binding protein
CDK	cyclin-dependent kinase
c-Myc	cellular Myc
COMT	catechol-O-methyltransferase
CoREST	co-repressor for element-1-silencing transcription factor
CpG	cytosine–phosphate–guanine
CSC	cancer stem cell
DNMT	DNA methyltransferase
DP	dimerization partner
E2F	transcription factor family
EGFR	epidermal growth factor receptor
EMT	epithelial–mesenchymal transition
ERs/ER/ERα	estrogen receptors/estrogen receptor/estrogen receptor alpha
ER+	estrogen receptor-positive
ErbB2	Erb-B2 receptor tyrosine kinase 2
ERK	extracellular signal-regulated kinase
ESR1	estrogen receptor 1 gene
FOXA1	Forkhead Box A1
FSH	follicle-stimulating hormone
G1/S	transition from G1 phase to S phase of the cell cycle
GATA3	GATA-binding protein 3
GST	glutathione S-transferase
HATs	histone acetyltransferases
HDACi	histone deacetylases inhibitors
HDACs	histone deacetylases
HER2	human epidermal growth factor receptor 2
HIF-1α	hypoxia-inducible factor-1α
HMTs	histone methyltransferases
HR+	hormone receptor-positive
HSP90	heat-shock protein 90
IARC	International Agency for Research on Cancer
IGFR	insulin-like growth factor receptor
Ki-67	proliferation marker protein
LH	luteinizing hormone
MAPK	mitogen-activated protein kinase
MeCP2	methyl-CpG-binding protein 2
MMP1	matrix metalloproteinase-1
mSin3	mammalian Sin3 corepressor
mTOR	mechanistic target of rapamycin
MuvB	multi-vulval class B complex
N-CoR	nuclear receptor corepressor
NF-κB	nuclear factor-κB
NuRD	nucleosome remodeling and deacetylase
OS	overall survival
p21^WAF1/CIP1	cyclin-dependent kinase inhibitor 1 = CDKN1A
p53	protein p53
PARP	poly(ADP-ribose) polymerase.
PCAF	CBP-associated factor
PFS	progression-free survival
PI3K	phosphoinositide 3-Kinase
PR	progesterone receptor
Rb	retinoblastoma protein
RB1	RB transcriptional corepressor 1
RB-like	retinoblastoma-like proteins p107/RBL1 and p130/RBL2
ROS	reactive oxygen species
RUNX3	runt-related transcription factor 3
SATB1	special AT-rich sequence-binding protein 1
SBK1	SH3 domain-binding kinase 1
SERD	selective estrogen receptor degrader
SERMs	selective estrogen receptor modulators
SHBG	sex hormone-binding globulin
Sin3 proteins	switch-independent 3 proteins
SIRT	sirtuins
Smad7	SMAD family member 7
SMRT	silencing mediator for retinoid and thyroid hormone receptors
Slug	snail family transcriptional repressor 2
Snail	snail family transcriptional repressor 1
SP1	specificity protein 1
STAT3	signal transducer and activator of transcription 3
TNF-α	tumor necrosis factor-α
TP53	tumor protein p53
TSA	trichostatin A
VEGF	vascular endothelial growth factor
VEGFR-2	vascular endothelial growth factor receptor 2
WHO	World Health Organization
ZEB1	zinc finger E-box-binding homeobox 1
